# Plasma DR-CALUX Bioactivity and Concurrent Clinical Biochemistry: A Secondary Cross-Sectional Analysis of SPES

**DOI:** 10.3390/ijms272110017

**Published:** 2026-11-08

**Authors:** Pellegrino Cerino, Elvira De Rosa, Maria Triassi, Paolo Montuori, Carlo Buonerba

**Affiliations:** 1 Department of Public Health, University of Naples “Federico II”, Via Sergio Pansini 5, 80131 Naples, Italy; 2 Department of Human Sciences and Quality of Life Promotion, San Raffaele University, 00166 Rome, Italy

**Keywords:** aryl hydrocarbon receptor, DR-CALUX, human biomonitoring, thyroid hormones, liver chemistries, persistent organic pollutants, lipid adjustment, exploratory analysis

## Abstract

Plasma bioassays provide functional measures of environmental mixtures whose relationship with clinical biochemistry warrants investigation. We examined dioxin-responsive chemically activated luciferase gene expression (DR-CALUX) bioactivity in the 600-participant intensive-biomonitoring subgroup of SPES in Campania, Italy. Quantifiable bioactivity was available for 594 participants, of whom 592 entered the principal analyses. Mixed models assessed the lipid-standardised DR-CALUX index in relation to two primary thyroid outcomes, nine secondary outcomes, and an exploratory panel of 54 clinical markers. Per index doubling, thyroid-stimulating hormone differed by −7.23% (95% CI −14.96 to 1.21; Holm-adjusted *p* = 0.182), and free thyroxine differed by −0.0047 ng/dL (−0.0233 to 0.0139; adjusted *p* = 0.621). Alanine and aspartate aminotransferase activities were lower by 9.33% (−14.22 to −4.17) and 8.01% (−12.31 to −3.50), respectively; both met the hepatic-family Benjamini–Hochberg criterion. Inverse estimates persisted with separate lipid adjustment and attenuated but remained inverse after restriction using common laboratory-report upper thresholds for both aminotransferases. All 54 global Benjamini–Yekutieli-adjusted *p* values exceeded 0.05; only the aminotransferases met the Benjamini–Hochberg comparison. These secondary aminotransferase associations merit further investigation of their biological basis and clinical significance. The primary thyroid findings remain inconclusive.

## 1. Introduction

Circulating environmental mixtures can be characterised by their chemical constituents and by the biological activity they elicit in a defined assay. These measurements address different aspects of internal exposure. Chemical panels quantify selected compounds, whereas functional assays can detect a shared response to material recovered from a biospecimen, including constituents beyond the individually quantified chemical panel. The blood exposome framework has encouraged the linking of such exposure measurements with biological phenotypes [1]. A central question is whether a functional signal in blood corresponds to differences in clinical biochemistry, and how those associations should be interpreted when the assay response is distinct from organ function in the participant.

Dioxin-responsive chemically activated luciferase gene expression (DR-CALUX) measures activity relative to a 2,3,7,8-tetrachlorodibenzo-p-dioxin (TCDD) reference in an aryl hydrocarbon receptor (AhR) reporter system. AhR is a ligand-activated transcription factor that regulates gene expression through its nuclear translocator and DNA response elements [2,3]. Early work adapted CALUX to extracted plasma, and subsequent studies applied the assay to pooled human sera [4,5]. The response integrates activity from compounds retained by sample preparation. It therefore differs from chemical toxic equivalents (TEQs), which are calculated from individually measured congener concentrations and assigned relative potencies.

The relationship between functional and chemical measurements requires empirical assessment. In pooled human sera from the Flemish Environment and Health Study, CALUX-derived and chemically calculated equivalents were correlated, but the strength of correlation varied between regions and their mean values differed substantially [5]. In older adults from the PIVUS cohort, raw-serum AhR activity was associated with chemically measured PCBs and toxic equivalents [2]. However, the raw-serum preparation used in that study differs from an extracted and cleaned DR-CALUX fraction. Such differences matter when translating evidence across studies: the compounds reaching the reporter cells, their availability and the assay conditions help determine the measured response. Associations with clinical measurements should consequently be interpreted for the assay and preparation actually used.

Thyroid regulation is a plausible target of dioxin-related bioactivity. Experimental TCDD exposure altered offspring thyroxine and hepatic hormone metabolism pathways in AhR-competent mice, with corresponding responses absent in AhR-null animals [6]. Human studies have produced differing results across populations and thyroid measurements, including lower total thyroxine after TCDD exposure in Seveso women, higher thyroid-stimulating hormone (TSH) in highly exposed Ranch Hand veterans, and higher free thyroid hormones in Vietnamese airbase workers [7,8,9]. A maternal-serum bioassay study reported null findings for dioxin-like activity and thyroid hormones during the second trimester [10]. This combination of biological plausibility and inconsistent human findings motivated the prioritisation of thyroid outcomes in SPES.

TSH and free thyroxine (FT4) were selected as complementary indicators of thyroid regulation: TSH reflects pituitary feedback, whereas FT4 measures circulating free hormone. Between-person associations reflect physiological feedback together with individual differences in thyroid regulation. Thyroid concentrations show substantial individual variation, and the TSH–FT4 relationship is nonlinear and differs by age and sex [11,12]. Assessing both markers, with multiplicity-controlled tests, therefore provides a fuller description of the thyroid phenotype. Analyte-specific findings in Anniston and the absence of adjusted associations in Brescia further illustrate the importance of phenotype definition when comparing environmental studies [13,14].

Hepatic biochemistry offers a related domain because the liver participates in xenobiotic processing and thyroid hormone metabolism. Routine markers describe several processes: aminotransferase activities principally indicate cellular injury, alkaline phosphatase and gamma-glutamyltransferase can reflect hepatobiliary processes, and bilirubin and circulating proteins provide additional physiological information. Epidemiological associations between persistent organic pollutants and hepatic markers have been reported in NHANES and PIVUS, although the pollutants and biochemical patterns differed [15,16]. Thyroid and hepatic measurements thus have a biological rationale for study alongside AhR-responsive bioactivity. Each marker reflects a distinct physiological process, and the clinical meaning of lower circulating enzyme activity requires specific evaluation.

Dioxins, furans and dioxin-like PCBs are lipophilic and partition into circulating lipids. A blood measurement can therefore vary with the amount of lipid carrying these compounds, as well as with their lipid-related burden. Expressing extracted dioxin-like bioactivity relative to lipid content provides a biological rationale for between-person comparison [17,18]. We selected a lipid-standardised index as the primary exposure on that basis. Because circulating lipids also relate to metabolic and hepatic phenotypes, the alternative exposure specifications were defined to assess the consequences of this choice.

SPES combined biospecimens, clinical measurements and questionnaire information in adults recruited across environmental-impact areas in Campania, southern Italy [19,20,21]. A subgroup of 600 of the 4205 enrolees underwent intensive biomonitoring. Published SPES work has examined serum trace elements in relation to lipid and blood-count measurements [22]; the present study concerns plasma DR-CALUX bioactivity in this selected subgroup.

We examined whether DR-CALUX bioactivity was associated with concurrent clinical biochemistry, prioritising TSH and FT4, evaluating liver-related and additional thyroid markers as secondary outcomes, and exploring the complete eligible non-lipid clinical panel. The analysis aimed to describe the magnitude and consistency of biochemical associations and place the focused thyroid and hepatic findings in the context of the wider available measurements.

## 2. Results

### 2.1. Analytical Population and Exposure Distribution

Quantifiable DR-CALUX results were available for 594 of the 600 selected participants; 592 had complete covariate data for the principal analyses. The analytical population represented 11 sampling clusters and 63 municipalities. Among participants with quantified bioactivity, median age was 34 years (interquartile range, IQR, 27–42), median body mass index (BMI) was 24.44 kg/m^2^ (22.41–27.34), and 299 (50.3%) were women. Table 1 describes this population and the principal analytical sample.

The median lipid-standardised DR-CALUX index among the 594 participants with quantified bioactivity was 42.22 (IQR 33.54–53.52). Outcome-specific sample sizes in the exploratory panel ranged from 562 to 592 because of missing laboratory measurements. Among the 592 participants in the principal analyses, median ALT was 35 U/L (IQR 30–40) and median AST was 23 U/L (19–28). The intervals printed in an available SPES laboratory report for an adult man (ALT 0–59 U/L; AST 0–40 U/L) were used as common descriptive benchmarks, with their clinical applicability qualified by the adult-male source report. ALT was within the benchmark in 573 participants (96.8%), AST was within the benchmark in 574 (97.0%), and both were within the benchmark in 569 (96.1%). Nineteen participants exceeded the ALT upper limit, 18 exceeded the AST upper limit, and 14 exceeded both; 23 exceeded at least one. Table S15 gives the descriptive counts.

The post hoc descriptive comparison used the 592 participants in the principal DR-CALUX analyses and 3589 SPES participants outside the original intensive-CALUX subgroup, identified within the established 4188-person analytical reference cohort (Table S16). Current smoking was recorded in 2/592 (0.34%) versus 1274/3539 (36.00%), consistent with the intended subgroup restriction. Recent/current medication use was reported by 126/590 (21.36%) versus 1251/3587 (34.88%); 235/592 (39.70%) versus 2156/3589 (60.07%) resided in high-impact areas. Age and several biochemical distributions were more similar. Percentages use the available denominator for each variable.

### 2.2. Primary Thyroid and Secondary Outcomes

Both primary thyroid Holm-adjusted *p* values exceeded 0.05 (Table 2). A doubling of the lipid-standardised DR-CALUX index corresponded to a −7.23% difference in TSH (95% CI −14.96 to 1.21; adjusted *p* = 0.182). The FT4 difference was −0.0047 ng/dL (95% CI −0.0233 to 0.0139; adjusted *p* = 0.621). Free triiodothyronine (FT3), the secondary thyroid outcome, differed by 0.0291 pg/mL (95% CI −0.0337 to 0.0919; *p* = 0.363).

Within the six-outcome enzyme/bilirubin family, ALT, AST, alkaline phosphatase (ALP) and direct bilirubin met the Benjamini–Hochberg (BH) threshold. ALT was lower by 9.33% (95% CI −14.22 to −4.17; adjusted *p* = 0.00195) and AST by 8.01% (−12.31 to −3.50; adjusted *p* = 0.00195). ALP was lower by 5.22% (−9.46 to −0.78; adjusted *p* = 0.0325), while direct bilirubin was higher by 9.73% (1.85 to 18.22; adjusted *p* = 0.0295). Gamma-glutamyltransferase (GGT) and total bilirubin had family-adjusted *p* values above 0.05. Albumin and pseudocholinesterase also had *p* values above the separate protein-related BH threshold. Table 2 reports all primary and secondary estimates.

### 2.3. Sensitivity Analyses

**Figure 1:**
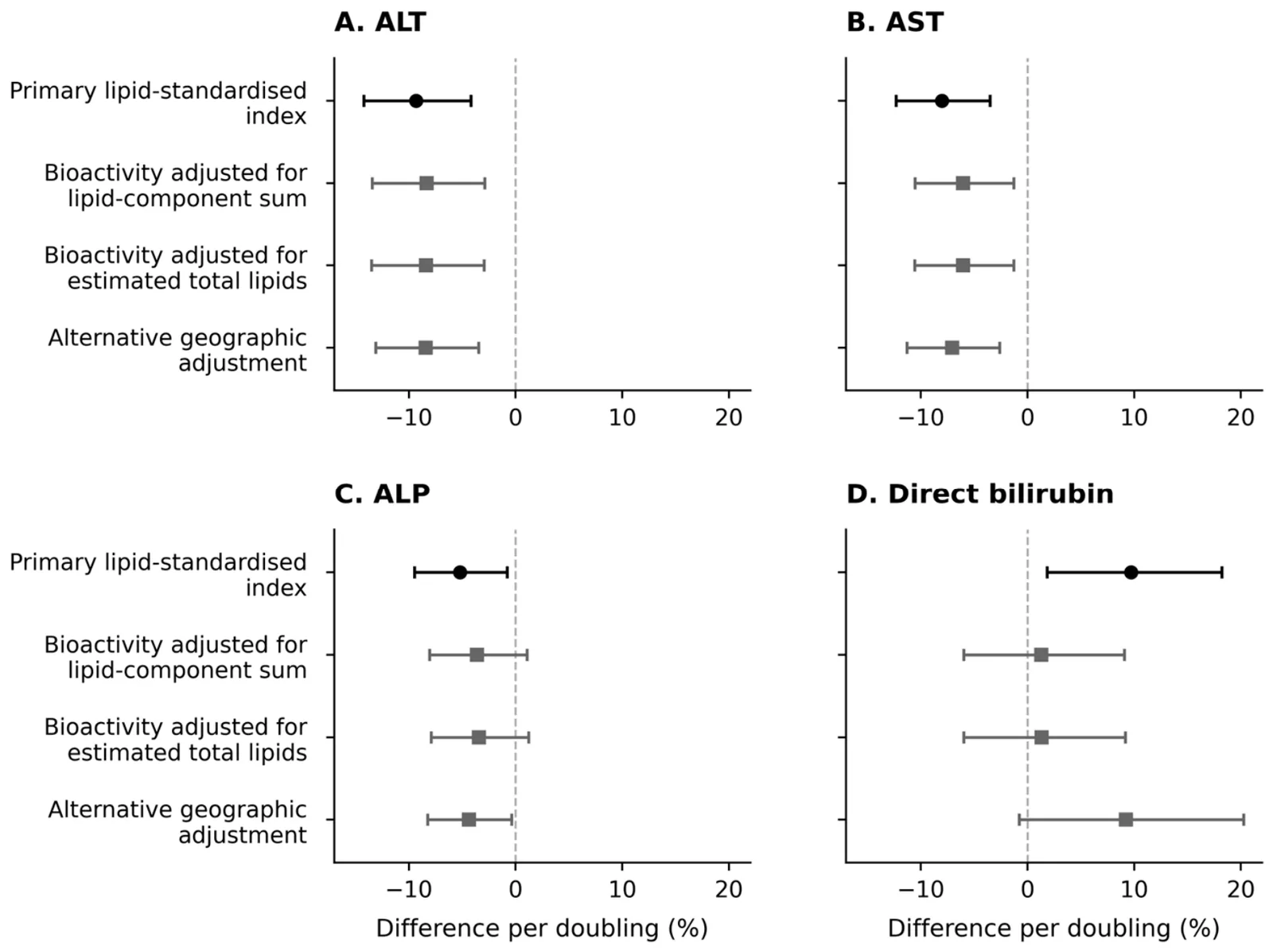
Associations for the four hepatic outcomes meeting the family-specific BH criterion under the primary model and three sensitivity specifications. (**A**) Alanine aminotransferase (ALT); (**B**) aspartate aminotransferase (AST); (**C**) alkaline phosphatase (ALP); (**D**) direct bilirubin. Points and bars show adjusted estimates and pointwise 95% confidence intervals. The primary and geographic models estimate a doubling of the lipid-standardised index. Models adjusting separately for the lipid-component sum or estimated total lipids estimate a doubling of bioactivity at fixed lipids. All models shown use 592 participants. The complete outcome panel and sensitivity results are provided in the Supplementary Materials.

The inverse ALT and AST associations persisted when bioactivity and lipids entered as separate model terms. With estimated total lipids, ALT differed by −8.38% (95% CI −13.51 to −2.96) and AST by −6.03% (−10.56 to −1.28); estimates were similar when adjustment used the lipid-component sum. The alternative geographic specification also retained inverse estimates for both enzymes (Figure 1). ALP and direct bilirubin were more sensitive to lipid representation: their estimates attenuated with separate lipid adjustment, and the confidence intervals included zero. Complete sensitivity estimates are provided in the Supplementary Materials.

ALT retained an inverse estimate with a pointwise confidence interval excluding zero in the primary model and all four alternative lipid specifications (Table 2 and Table S7). Without a lipid term, non-normalised bioactivity was associated with an ALT difference of −6.35% (95% CI −11.47 to −0.94). AST was more dependent on lipid handling: its corresponding estimate was −3.22% (−7.83 to 1.62). The alternative geographic model gave a TSH estimate of −8.70% (−16.14 to −0.61); interpretation of the primary thyroid test follows the original Holm adjustment. TSH estimates were closer to zero with separate lipid terms (−3.42% and −3.60%, both intervals including zero) and without a lipid term (0.03%) (Table S7).

Exploratory tests of exposure nonlinearity and sex interaction for TSH and FT4 had adjusted *p* values of at least 0.348. Thyroid spline curves and influence diagnostics are reported in the Supplementary Materials.

Among the 569 participants with both aminotransferases at or below the common upper benchmarks, estimates attenuated to −6.54% for ALT (95% CI −11.20 to −1.64) and −5.06% for AST (−8.56 to −1.44) per index doubling (Table S17). Thus, the inverse pattern extended to participants with both enzymes at or below the selected benchmarks.

In the previously specified restriction to 464 participants explicitly reporting no medication use during the preceding six months or currently, ALT and AST estimates were −11.98% (95% CI −17.67 to −5.90) and −10.34% (−15.21 to −5.19), respectively (Tables S7 and S8). This broad self-report provides limited detail on individual medicines.

The pooled exposure-by-area interaction tests provided limited evidence of heterogeneity: ALT F = 0.199, numerator df = 2, denominator df = 559.18, *p* = 0.8196, BH-adjusted *p* = 0.8196; AST F = 1.106, numerator df = 2, denominator df = 536.04, *p* = 0.3317, and BH-adjusted *p* = 0.6635 (Table S18). All area-specific point estimates were inverse. The low-impact area contained only two sampling clusters, limiting interpretation of its wider intervals.

Among the four focal original models, TSH had a boundary random-intercept variance; FT4, ALT and AST had positive variance estimates (Table S19). ALT and AST intraclass correlations, defined as random-intercept variance divided by random-intercept plus residual variance, were 0.0291 and 0.0143. The alternative geographic specification yielded −8.41% for ALT (95% CI −13.10 to −3.47) and −7.05% for AST (−11.30 to −2.60), with exposure-specific Satterthwaite df = 21.42. All 1332 ALT/AST participant-, municipality- and sampling-cluster-deletion refits were available and retained inverse coefficients (Table S20). Sampling-cluster deletions gave ALT estimates from −10.33% to −8.59% and AST estimates from −8.95% to −6.58%. One AST municipality-deletion fit and one AST sampling-cluster-deletion fit reached the variance boundary. These refits evaluated changes in the exposure coefficient; inferential tests remained those of the full models.

### 2.4. Exploratory Analysis of 54 Clinical Markers

The exploratory panel included the 11 primary and secondary outcomes and 43 additional markers spanning renal, electrolyte/mineral, glycaemic, iron-related, haematological, protein and endocrine measurements. All 54 principal models yielded estimable exposure contrasts.

All global Benjamini–Yekutieli (BY)-adjusted *p* values exceeded 0.05. ALT and AST alone met the parallel BH comparison, both with BH-adjusted *p* = 0.0176 and BY-adjusted *p* = 0.0805. All 43 additional outcomes had *p* values above both adjusted thresholds. Table 3 summarises the panel by domain; the Supplementary Materials report all effects, confidence intervals, unadjusted and adjusted *p* values, sensitivity estimates and model diagnostics.

## 3. Discussion

In this selected SPES subgroup, higher lipid-standardised DR-CALUX bioactivity was associated with lower ALT and AST activities, while both primary thyroid tests had Holm-adjusted *p* values above 0.05. The inverse aminotransferase estimates persisted under alternative lipid-adjusted and geographic specifications and attenuated but remained inverse after restriction using common upper benchmarks for both enzymes. ALP and direct bilirubin were more dependent on lipid representation. The aminotransferase pattern is therefore the main secondary finding for further investigation, with its statistical support interpreted within the original outcome hierarchy and broader exploratory panel.

The thyroid estimates differed in precision. The TSH interval ranged from approximately 15% lower to 1% higher per exposure doubling, remaining compatible with a moderate inverse association. The FT4 estimate was close to zero, with an interval of −0.0233 to +0.0139 ng/dL. Between-person thyroid measurements reflect individual set points as well as feedback, and the TSH–FT4 relationship varies with age and sex [11,12]. These complementary estimates provide limited support for a thyroid association and quantify the compatible mean differences at the achieved precision [23].

Previous thyroid studies show heterogeneous patterns. Foster and colleagues reported null findings in a maternal-serum bioassay study [10]. In Seveso women, TCDD was inversely associated with total T4 at one follow-up assessment, with null findings for TSH, FT4 and FT3 [7]. Anniston investigators reported associations of some PCB groupings with total T3, alongside null findings for TSH, total T4 and FT4 [13], whereas Vietnamese airbase workers showed positive associations with FT3 and FT4 [9]. Exposure composition, timing, physiological state and assay preparation are important distinctions when comparing these findings with SPES.

Experimental evidence connects thyroid regulation with hepatic metabolism: TCDD altered offspring thyroxine and hepatic glucuronosyltransferase expression through AhR-dependent pathways in mice [6], and other experiments demonstrate AhR-dependent hepatic responses [24,25]. This biology supports examining both domains, while the SPES measurements characterise concurrent biochemical associations. Hormone clearance and tissue-level receptor activity would require dedicated measurements.

The inverse aminotransferase direction contrasts with the association between PCB concentrations and elevated ALT in NHANES [15]. The FINRISK study examined persistent pollutants in relation to liver-related biomarkers and subsequent clinically recorded liver disease [26]. Yet hepatic findings vary by chemical and marker: the NHANES analysis reported null findings for ALT elevation in relation to the examined PCDD/F measurements, while PIVUS reported positive mono-ortho-PCB–bilirubin and inverse pesticide–ALP associations [15,16]. These studies assessed chemical concentrations, elevated enzymes or subsequent disease; SPES examined continuous biochemical variation in relation to extracted-plasma bioactivity. Differences in population, metabolic health and mixture composition may contribute to the differing patterns.

The exposure measure is important to this comparison. DR-CALUX integrates the response of an extracted plasma fraction, with cleanup and assay conditions determining the contributing mixture [3,4]. Human-plasma validation studies reported moderate correspondence with chemical TEQs: r = 0.64 in 341 samples and Spearman r = 0.66 in a 15-person paired subset [27,28]. The larger series also showed proportional systematic disagreement. These protocol-specific external results support complementary interpretations of bioactivity and chemical burden. SPES therefore contributes evidence about the functional mixture signal, while attribution to particular constituents remains unresolved.

Persistent lipophilic dioxin-like compounds distribute with circulating lipids. Lipid normalisation therefore expresses recovered activity relative to the circulating lipid compartment [17,18,29]. The primary index follows the historical SPES clinical lipid-component framework [30]. Extraction prepares the tested material while its original lipid distribution remains relevant. Circulating lipids also relate to metabolic and hepatic characteristics, making alternative exposure representations informative. ALT remained inverse across all five specifications; AST was more sensitive, with an interval including zero when lipids were omitted. Direct bilirubin attenuated markedly with separate lipid terms, limiting a consistent physiological interpretation (Table S7).

Most participants had aminotransferase values at or below the common laboratory-report benchmarks. The inverse estimates attenuated but remained evident in the joint benchmark-restricted subset, extending the pattern beyond participants with elevated values. Joint outcome-range restriction changes the population and can itself affect the estimates. These routine liver chemistries characterise biochemical variation; assessment of liver function and underlying disease requires broader clinical information [31].

Longitudinal studies have reported a counterintuitive association: lower ALT activity, including values within reference ranges, has been associated with higher subsequent all-cause mortality in a middle-aged screening cohort and NHANES III [32,33]. In older InCHIANTI participants, low ALT and AST were associated with frailty, sarcopenia and disability, alongside nonlinear mortality relationships [34]. These observations provide hypothesis-generating context for investigating physiological state and body composition, which BMI captures only partly, as correlates of the SPES pattern. AST also has extrahepatic sources; experimental exercise-related aminotransferase elevations illustrate a muscle contribution [35]. The SPES estimates describe between-person biochemical differences at a single assessment among adults aged 20–50 years. Repeated measurements and clinical follow-up could clarify their relevance to subsequent health.

AhR-related regulation of hepatic processes is another possible explanation for the pattern. Establishing an effect on aminotransferase synthesis or release would require measurements of tissue receptor activity, enzyme expression and turnover. Differences in metabolic state, body composition, treatment indication and other confounders remain alternative explanations. The clinical significance of the inverse associations therefore warrants investigation beyond routine enzyme measurements.

The wider clinical panel helps define the scope of the findings. ALT and AST met the original hepatic-family BH criterion and the global BH comparison; all global BY-adjusted *p* values exceeded 0.05 [36,37]. They remain secondary candidate associations, with the broader screen extending the biochemical context within the same analytical population. Dhooge and colleagues reported that higher serum dioxin-like activity measured by DR-CALUX was associated with lower total and free testosterone in young Flemish men, in analyses restricted to activity above a study-specific threshold [38]. Their male-only population and threshold-based analysis differ from the pooled, sex-adjusted SPES models. The original family-specific corrections and global exploratory assessment answer distinct multiplicity questions.

Generalisability reflects both recruitment from the community-based SPES cohort and the selection criteria for intensive biomonitoring. Selection across age, sex, BMI and environmental-impact areas, together with intended restrictions on smoking, alcohol abuse and chronic-disease medication, defines the population context of these findings. The estimates are most directly applicable to adults with characteristics similar to those of the analysed subgroup. The comparison of 592 analytical participants with 3589 nonselected cohort members describes the resulting differences in sample composition (Table S16). Initial selection and analytical completeness jointly shape this population. The cross-sectional measurements describe contemporaneous exposure–biochemistry associations, with their temporal ordering remaining uncertain.

The common adjustment set and targeted sensitivities address measured demographic and lifestyle factors. The broader SPES questionnaires captured self-reported dyslipidaemia, diabetes, occupational exposure and treatment histories, including current oral-contraceptive and menopausal-hormone use. These histories remained outside the principal adjustment set. Their limited detail on current disease severity, drug class, dose, adherence, occupational intensity and endocrine conditions at blood collection restricts the interpretability of further disease- or treatment-specific adjustment. Chronic liver disease was incompletely characterised. The broad-medication restriction assesses reported recent/current nonuse in a different subset and provides a sensitivity check within that scope. The analyses retained the original adjustment set and targeted sensitivities; reproductive state, menstrual phase, collection timing and hormone-specific treatment remain sources of residual variation. Unreported disease, treatment and physiological differences may consequently influence both the hepatic and endocrine estimates.

Statistical uncertainty also reflects the eleven-cluster design, which provides limited information about between-cluster variance. TSH had a boundary random-intercept estimate, and residual-distribution departures were apparent, particularly for AST (Tables S13 and S19). Sensitivity and deletion checks support coefficient stability under the examined specifications, while interval calibration remains conditional on covariance assumptions, including possible dependence between municipalities within sampling clusters. Area-specific inference is especially limited by the two low-impact clusters. Missing laboratory measurements, particularly blood counts, further restrict comparisons across domains.

The inverse aminotransferase pattern merits independent assessment with repeated bioactivity and biochemical measurements. Combining these with measures of metabolic health, body composition and targeted hepatic injury, such as cytokeratin-18 assessed in Anniston [39], could help distinguish exposure-related biology from differences in physiological state or residual confounding. Longitudinal follow-up could assess whether the observed aminotransferase pattern is associated with subsequent health outcomes.

## 4. Materials and Methods

### 4.1. Study Design and Participants

We conducted a secondary cross-sectional analysis of the 600-participant intensive-biomonitoring subgroup of the Exposure Study on Susceptible People (SPES). The parent programme recruited adults from municipal registries across environmental-impact areas in Campania, Italy, during 2016–2017 and combined questionnaires, clinical assessment and biospecimen collection. Published reports describe the recruitment framework and implementation [19,20]. The present analysis used archived subgroup records and unweighted models.

The subgroup comprised 600 of the 4205 SPES enrolees, selected across sex-by-age strata and environmental-impact areas. The design allocated 240 participants to high-impact areas, 240 to medium-impact areas and 120 to low-impact areas, with BMI stratification and intended restrictions concerning smoking, alcohol abuse, medication for chronic disease and higher obesity classes [30]. This study used the existing subgroup and its DR-CALUX measurements. Eligibility for the present analyses was based on available exposure, outcome and covariate data; the two recorded current smokers were retained.

To assess the generalisability of the principal findings, we compared the 592 participants included in the principal DR-CALUX analyses with 3589 participants in the established 4188-person SPES analytical reference cohort outside the original 600-person intensive-biomonitoring subgroup [21]. Exact one-to-one linkage identified both groups. The comparison group was restricted to participants outside that original subgroup. Common source fields, variable-specific available denominators and standardised mean differences were used (Table S16; Supplementary Materials S1). This post hoc comparison was unweighted and descriptive, with group differences summarised using standardised differences.

The intensive-biomonitoring programme also measured ERα-CALUX and PAH-CALUX activity and chemically quantified PCDD/Fs and PCBs. Related SPES studies address endocrine and reproductive phenotypes using ERα-CALUX, vascular, one-carbon and haematologic phenotypes using PAH-CALUX [40], and associations with chemically quantified pollutants. These studies share participants and some clinical measurements with the present DR-CALUX analysis; their findings therefore constitute related evidence from overlapping populations. The present models concern DR-CALUX bioactivity as the exposure of interest.

The thyroid and hepatic analysis plan was fixed before the first association run. The complete broader-panel specification was finalised after completion of the initial analyses and before fitting the additional associations. The broader panel is reported as exploratory; the study followed an internally documented analysis plan. During peer review, additional selection, joint-benchmark, area-interaction and influence analyses were specified with the original findings already known; they retained the original outcome hierarchy and exposure definition.

Participants with linked, positive, quantifiable DR-CALUX results were eligible. Outcome-specific analyses used complete exposure, outcome and covariate information. Participant flow is reported in Table S1.

### 4.2. Biospecimens, DR-CALUX and Lipid Representation

Clinical specimens and stored CALUX specimens were collected at SPES enrolment. Heparinised plasma aliquots were stored at −80 °C and shipped frozen to BioDetection Systems (BDS), Amsterdam, the Netherlands, which performed the DR-CALUX analyses. Clinical lipid concentrations were measured in serum. Plasma extracts underwent acid-silica cleanup before a 24 h exposure in the H4IIe AhR-responsive luciferase reporter assay against a TCDD calibration curve. Calibration interpolation used GraphPad Prism 5.03 (GraphPad Software, San Diego, CA, USA). The reporter principle and its application to blood plasma have been described previously [3,4]. Procedure blanks and reference plasma controls accompanied analysis. Final laboratory-reported results and quantification status were used in the present study. The exposure therefore represents assay-defined dioxin-like bioactivity in the prepared extract.

Lipid normalisation was selected because the persistent lipophilic compounds targeted by DR-CALUX partition into circulating lipids, providing a biological basis for expressing recovered bioactivity relative to lipid content. The original SPES institutional report used lipid-based DR-CALUX reporting [30]. The present analysis retained the historical clinical component-sum index, specified before association fitting: R = B/[(TC + TG + PL)/100,000], where B denotes non-normalised bioactivity on the laboratory-reported numerical scale and TC, TG and PL denote serum total cholesterol, triglycerides and phospholipids in mg/dL. The factor 100,000 preserves the source index scale, with the per-doubling contrast invariant to this common factor. The model represented exposure by log_2_(R) alone. This operational index characterises assay-defined mixture bioactivity; comparisons concern relative associations on the specified exposure scale.

The alternative lipid models evaluated the statistical implications of the primary biologically motivated exposure definition. They replaced the ratio with log_2_(B) and log_2_(lipids) as separate, freely estimated terms, used Akins-estimated total lipids as an alternative denominator, or omitted lipid adjustment. The lipid terms used the historical component sum or Akins-estimated total lipids. Each alternative replaced the primary exposure specification [17,18,41,42]. Full formulas are given in Supplementary Materials S3.

### 4.3. Clinical Outcomes and Covariates

Clinical biochemical and haematological measurements were obtained at enrolment through routine testing in laboratories certified for human specimens. Available SPES laboratory documentation describes IFCC methods at 37 °C for ALT and AST. The intervals in an available report for an adult man (ALT 0–59 U/L; AST 0–40 U/L) were used as common descriptive benchmarks for all participants (Table S15), with clinical applicability qualified by the source report. The original models retained continuous measurements; the reviewer-requested restriction is specified in Section 4.5. TSH (mIU/L) and FT4 (ng/dL) were the two primary outcomes. FT3 was a secondary outcome; ALT, AST, GGT, ALP, total bilirubin and direct bilirubin formed a six-outcome secondary family, and albumin and pseudocholinesterase formed a two-outcome family. TSH, the six enzyme/bilirubin markers and pseudocholinesterase were natural-log-transformed; FT4, FT3 and albumin retained their original scales. Each primary thyroid outcome was assessed against its own Holm-adjusted criterion.

The clinical inventory comprised 76 source fields and defined 54 outcomes, including the 11 primary and secondary measurements. Eligibility required an interpretable quantitative measurement available for at least 80% of the 594 participants with quantifiable DR-CALUX. Lipids, duplicate representations and selected deterministic redundancies were excluded. Twenty-two outcomes used natural-log transformations and 32 retained their original scales. The complete inventory, transformations and exclusions are reported in Supplementary Materials S4 and the Supplementary Tables.

Units were harmonised where necessary, and a single albumin measurement was used consistently. Total and direct bilirubin were retained; indirect bilirubin was excluded as a redundant representation. Supplementary Materials S4 describes the data handling.

Models adjusted for age, sex, BMI, recorded current smoking, log(1 + alcohol in g/day) and environmental-impact area. Age and BMI used three-knot restricted cubic splines, with knots at the 10th, 50th and 90th percentiles of the 594-person exposure-qualified population reused across models. The common covariate set was specified on substantive grounds and applied to each outcome separately [43]. A medication sensitivity analysis was restricted to participants reporting no medication use during the previous six months or currently.

Analyses used observed, outcome-specific complete cases and quantified exposures. Sample sizes ranged from 562 to 592, with eligibility assessed separately for each outcome. Below-quantification-limit exposures remained excluded. Endocrine models pooled men and women with sex adjustment and a common exposure slope.

### 4.4. Statistical Models and Multiplicity

Each outcome was modelled separately using log_2_(R), the specified outcome transformation, the common covariates and a sampling-cluster random intercept. Models used restricted maximum likelihood with Kenward–Roger inference for two-sided tests and pointwise 95% confidence intervals [44,45]. Effects were expressed as adjusted mean differences for untransformed outcomes and as 100 × [exp(β) − 1] for log-transformed outcomes, where β is the exposure coefficient. Thus, estimates describe differences per doubling of the index. Convergence and estimability were checked; converged fits with a boundary random-intercept variance were retained and flagged.

Holm adjustment applied to TSH and FT4. Separate BH adjustments applied to the six enzyme/bilirubin outcomes and the two protein-related outcomes; FT3 was assessed separately using its unadjusted *p* value. All 54 outcomes formed one global exploratory family, with BY-adjusted *p* ≤ 0.05 as the principal criterion and BH-adjusted values as a less conservative comparison. Each multiplicity procedure applies to its specified family in this DR-CALUX analysis. Confidence intervals were pointwise, and complete results were reported irrespective of statistical support [36,37,46].

### 4.5. Sensitivities, Diagnostics and Reproducibility

Sensitivity analyses examined lipid representation, geographic adjustment, covariate specification and selected participant restrictions. Three comparisons were applied uniformly to all 54 outcomes: separate adjustment for the lipid-component sum; separate adjustment for estimated total lipids; and sampling-cluster fixed effects with municipality-clustered CR2/Satterthwaite inference [47]. Separate lipid models estimate a doubling of bioactivity at fixed lipids; the primary and geographic models estimate a doubling of the lipid-standardised index. Sensitivity analyses focused on estimates and pointwise intervals. Supplementary Materials S8 specifies each comparison and its outcome scope.

The original exploratory exposure-spline and sex-interaction analyses were confined to TSH and FT4, with BH correction performed within three two-outcome families. Original participant- and municipality-deletion diagnostics also concerned TSH and FT4 (Table S12). All 54 models underwent residual, convergence and estimability checks; observations flagged by residual magnitude were retained.

The reviewer-requested sensitivity analyses retained the original transformations, covariates, spline knots and REML/Kenward–Roger implementation. ALT and AST models were repeated after requiring 0 < ALT ≤ 59 U/L and 0 < AST ≤ 40 U/L simultaneously (n = 569; Table S17). These thresholds were used as common descriptive benchmarks based on the adult-male source report.

One pooled exposure-by-environmental-impact-area model was fitted for both ALT and AST. The two interaction coefficients were tested jointly using a two-degree-of-freedom Kenward–Roger F test; BH correction covered the two omnibus tests. All area-specific slopes were obtained as contrasts of the pooled models with the full adjusted covariance matrix and pointwise intervals, and area-specific participant, cluster and exposure support was reported (Table S18). The joint-benchmark sensitivity remained estimation-focused.

For ALT and AST, the original models were refitted after omitting each participant, municipality and sampling cluster in turn, retaining the same exposure definition and spline knots. These 1332 refits assessed coefficient changes, availability and boundary variance estimates. Kenward–Roger covariance matrices, confidence intervals and tests were calculated for the full models only. Coefficient changes were divided by the full-model standard error as a descriptive scaled shift. Variance components and residual summaries are reported in Table S19, and deletion summaries in Table S20. Thyroid deletion analyses considered participants and municipalities only (Table S12).

Association analyses were performed in R 4.6.1 (R Foundation for Statistical Computing, Vienna, Austria) using lme4 2.0-6, pbkrtest 0.5.5 and clubSandwich 0.7.0. Aggregate results are reported in the Supplementary Materials. Data-free analytical code and supporting aggregate tables are supplied separately for editorial and peer review in Non-published Material.zip. Reporting follows STROBE and STROBE-ME [48,49,50]. Claude Opus 5 (Anthropic, San Francisco, CA, USA) and ChatGPT (OpenAI, San Francisco, CA, USA; GPT-6) were used for language editing and R-code generation. The R workflow generated the association estimates; synthetic tests and independent checks of source data and numerical outputs supported verification, with the authors responsible for the analytical decisions and interpretation.

## 5. Conclusions

Higher lipid-standardised plasma DR-CALUX bioactivity was associated with lower ALT and AST activities in the selected SPES subgroup. The inverse estimates were retained under alternative lipid-adjusted specifications and attenuated, but remained inverse, after restriction using common upper benchmarks for both enzymes. Their biological basis and clinical significance remain uncertain. Both primary thyroid Holm-adjusted *p* values and all global BY-adjusted *p* values across 54 markers exceeded 0.05. Independent studies incorporating repeated biochemical measurements and clinical outcomes are needed to establish the clinical relevance of these secondary candidate associations.

## Figures and Tables

**Table 1:** Characteristics of participants with quantified DR-CALUX and of the principal analytical population.

Characteristic	Quantified DR (n = 594)	Principal Models (n = 592)
Age (years)	34 [27, 42]	34 [27, 42]
BMI (kg/m^2^)	24.44 [22.41, 27.34]	24.44 [22.43, 27.34]
Alcohol (g/day)	0.15 [0.15, 6.07]; n = 592	0.15 [0.15, 6.07]
Non-normalised DR-CALUX (source scale)	0.227 [0.183, 0.291]	0.227 [0.183, 0.290]
Lipid-standardised DR-CALUX index	42.22 [33.54, 53.52]	42.22 [33.64, 53.39]
Total cholesterol (mg/dL)	185.0 [163.0, 210.0]	185.0 [163.0, 210.0]
Free cholesterol (mg/dL)	34.4 [28.5, 41.6]	34.4 [28.5, 41.6]
Triglycerides (mg/dL)	85.0 [68.0, 114.8]	85.0 [68.0, 115.0]
Phospholipids (mg/dL)	254.0 [229.0, 282.0]	254.0 [229.0, 282.0]
Lipid-component sum (mg/dL)	532.5 [475.0, 602.0]	532.5 [474.8, 602.2]
Estimated total lipids (mg/dL)	627.7 [566.3, 717.2]	627.7 [566.4, 717.0]
Women	299 (50.3%)	298 (50.3%)
Men	295 (49.7%)	294 (49.7%)
Recorded current smoking	2 (0.3%)	2 (0.3%)
Low-impact area	120 (20.2%)	120 (20.3%)
Medium-impact area	238 (40.1%)	237 (40.0%)
High-impact area	236 (39.7%)	235 (39.7%)
Recent/current medication: yes	126 (21.2%)	126 (21.3%)
Recent/current medication: no	466 (78.5%)	464 (78.4%)
Recent/current medication: unknown	2 (0.3%)	2 (0.3%)

Continuous values are median [IQR]; categorical values are n (%), using the column denominator. BMI, body mass index; DR-CALUX, dioxin-responsive chemically activated luciferase gene expression. Section 4.2 defines the lipid-standardised index and its numerical scale. Alcohol was available for 592 participants in both columns. Table S16 compares the same 592-person principal analytical sample with nonselected SPES participants. Its medication percentages use available responses, whereas this table uses the full column denominator and displays unknown responses separately.

**Table 2:** Primary and secondary associations per doubling of the lipid-standardised DR-CALUX index.

Outcome	Scale	Difference (95% CI)	Raw *p*	Adjusted *p*	Family
TSH	%	−7.23 (−14.96, 1.21)	0.0911	0.182	Holm, 2
FT4	ng/dL	−0.0047 (−0.0233, 0.0139)	0.621	0.621	Holm, 2
FT3	pg/mL	0.0291 (−0.0337, 0.0919)	0.363	0.363	Singleton
ALT	%	−9.33 (−14.22, −4.17)	0.00054	0.00195	BH, 6
AST	%	−8.01 (−12.31, −3.50)	0.00065	0.00195	BH, 6
GGT	%	−7.27 (−13.97, −0.04)	0.0488	0.0586	BH, 6
ALP	%	−5.22 (−9.46, −0.78)	0.0217	0.0325	BH, 6
Total bilirubin	%	5.25 (−2.44, 13.56)	0.186	0.186	BH, 6
Direct bilirubin	%	9.73 (1.85, 18.22)	0.0148	0.0295	BH, 6
Albumin	g/dL	−0.0434 (−0.0863, −0.0004)	0.0477	0.0743	BH, 2
Pseudocholinesterase	%	−2.27 (−4.71, 0.23)	0.0743	0.0743	BH, 2

All models: n = 592; 11 sampling clusters; 63 municipalities. Adjustment: age and BMI restricted cubic splines, sex, current smoking, log(1 + alcohol), environmental-impact area and sampling-cluster random intercept. REML with Kenward–Roger inference. Percentages are differences in conditional geometric means; other effects are mean differences in the stated units. Confidence intervals are pointwise. BH, Benjamini–Hochberg; CI, confidence interval; REML, restricted maximum likelihood. FT3 was assessed as a secondary singleton using its unadjusted *p* value. ALT, alanine aminotransferase; AST, aspartate aminotransferase; ALP, alkaline phosphatase; GGT, gamma-glutamyltransferase; FT3/FT4, free triiodothyronine/thyroxine; TSH, thyroid-stimulating hormone.

**Table 3:** Coverage and multiplicity results of the exploratory clinical panel.

Domain	Outcomes	Added	Model n	BY ≤ 0.05	BH ≤ 0.05
Electrolytes and minerals	5	5	592	0	0
Glucose metabolism	1	1	592	0	0
Haematology	15	15	562, 563	0	0
Hepatic biochemistry	6	0	592	0	2
Iron-related	3	3	592	0	0
One-carbon metabolism	1	1	592	0	0
Other endocrine	8	8	592	0	0
Other enzyme	1	1	592	0	0
Protein electrophoresis	6	6	592	0	0
Protein-related	2	0	592	0	0
Renal-related	3	3	592	0	0
Thyroid	3	0	592	0	0
All eligible markers	54	43	562–592	0	2

The 54 outcomes form one global family; domains are descriptive groupings. BY, Benjamini–Yekutieli, was the global criterion; BH was the comparison. Only ALT and AST met BH ≤ 0.05 (both 0.0176; BY 0.0805). Outcome-specific sample sizes differed because of missing measurements: most models used 592 participants, blood-count models used 563, and mean platelet volume used 562.

## Data Availability

Aggregate results and measurement definitions are reported in the Supplementary Materials. Data-free analytical code and supporting aggregate tables are supplied for editorial and peer review in Non-published Material.zip, separately from the public supplementary files. Reproducing participant-level models requires authorised access to the controlled study inputs. Individual clinical and biomonitoring records, laboratory reports and linkage files remain in controlled access under SPES privacy and institutional data-governance arrangements. Requests for controlled study data may be directed to the corresponding author, Carlo Buonerba (carbuone@hotmail.com), for consideration under the applicable access arrangements.

## Supplementary Materials

The following supporting information can be downloaded at: https://www.mdpi.com/article/10.3390/ijms272110017/s1. References [3,4,17,18,19,20,21,29,30,36,37,41,42,43,44,45,46,47] are cited in the Supplementary Materials.

## Abbreviations

AhR, aryl hydrocarbon receptor; ALP, alkaline phosphatase; ALT, alanine aminotransferase; AST, aspartate aminotransferase; BH, Benjamini–Hochberg; BMI, body mass index; BY, Benjamini–Yekutieli; CALUX, chemically activated luciferase gene expression; CI, confidence interval; CR2, bias-reduced cluster-robust covariance estimator; DR, dioxin-responsive; FT3, free triiodothyronine; FT4, free thyroxine; GGT, gamma-glutamyltransferase; IQR, interquartile range; PCB, polychlorinated biphenyl; PCDD/F, polychlorinated dibenzo-p-dioxin/dibenzofuran; PL, phospholipids; REML, restricted maximum likelihood; SPES, Exposure Study on Susceptible People; TC, total cholesterol; TCDD, 2,3,7,8-tetrachlorodibenzo-p-dioxin; TEQ, toxic equivalent; TG, triglycerides; TSH, thyroid-stimulating hormone.

## References

[1] Rappaport S.M., Barupal D.K., Wishart D., Vineis P., Scalbert A. (2014). The blood exposome and its role in discovering causes of disease. Environ. Health Perspect..

[2] Park W.H., Kang S., Lee H.K., Salihovic S., van Bavel B., Lind P.M., Pak Y.K., Lind L. (2017). Relationships between serum-induced AhR bioactivity or mitochondrial inhibition and circulating polychlorinated biphenyls (PCBs). Sci. Rep..

[3] Garrison P.M., Tullis K., Aarts J.M.M.J.G., Brouwer A., Giesy J.P., Denison M.S. (1996). Species-specific recombinant cell lines as bioassay systems for the detection of 2,3,7,8-tetrachlorodibenzo-p-dioxin-like chemicals. Fundam. Appl. Toxicol..

[4] Murk A.J., Leonards P.E.G., Bulder A.S., Jonas A.S., Rozemeijer M.J.C., Denison M.S., Koeman J.H., Brouwer A. (1997). The CALUX (chemical-activated luciferase expression) assay adapted and validated for measuring TCDD equivalents in blood plasma. Environ. Toxicol. Chem..

[5] Koppen G., Covaci A., Van Cleuvenbergen R., Schepens P., Winneke G., Nelen V., Schoeters G. (2001). Comparison of CALUX-TEQ values with PCB and PCDD/F measurements in human serum of the Flanders Environmental and Health Study (FLEHS). Toxicol. Lett..

[6] Nishimura N., Yonemoto J., Miyabara Y., Fujii-Kuriyama Y., Tohyama C. (2005). Altered thyroxin and retinoid metabolic response to 2,3,7,8-tetrachlorodibenzo-p-dioxin in aryl hydrocarbon receptor-null mice. Arch. Toxicol..

[7] Chevrier J., Warner M., Gunier R.B., Brambilla P., Eskenazi B., Mocarelli P. (2014). Serum dioxin concentrations and thyroid hormone levels in the Seveso Women’s Health Study. Am. J. Epidemiol..

[8] Pavuk M., Schecter A.J., Akhtar F.Z., Michalek J.E. (2003). Serum 2,3,7,8-tetrachlorodibenzo-p-dioxin (TCDD) levels and thyroid function in Air Force veterans of the Vietnam War. Ann. Epidemiol..

[9] Pham The T., Nishijo M., Phan Van M., Nguyen Minh P., Pham Ngoc T., Vu Thi H., Nguyen Van C., Tran Ngoc N., Do Minh T., Dao Duc L. (2024). Effects of dioxin exposure on reproductive and thyroid hormone levels and male sexual function in airbase military workers in Vietnam. Environ. Sci. Pollut. Res. Int..

[10] Foster W.G., Holloway A.C., Hughes C.L. (2005). Dioxin-like activity and maternal thyroid hormone levels in second trimester maternal serum. Am. J. Obstet. Gynecol..

[11] Andersen S., Pedersen K.M., Bruun N.H., Laurberg P. (2002). Narrow individual variations in serum T(4) and T(3) in normal subjects: A clue to the understanding of subclinical thyroid disease. J. Clin. Endocrinol. Metab..

[12] Hadlow N.C., Rothacker K.M., Wardrop R., Brown S.J., Lim E.M., Walsh J.P. (2013). The relationship between TSH and free T_4_ in a large population is complex and nonlinear and differs by age and sex. J. Clin. Endocrinol. Metab..

[13] Benson K., Yang E., Dutton N., Sjodin A., Rosenbaum P.F., Pavuk M. (2018). Polychlorinated biphenyls, indicators of thyroid function and thyroid autoantibodies in the Anniston Community Health Survey I (ACHS-I). Chemosphere.

[14] Zani C., Magoni M., Speziani F., Leonardi L., Orizio G., Scarcella C., Gaia A., Donato F. (2019). Polychlorinated biphenyl serum levels, thyroid hormones and endocrine and metabolic diseases in people living in a highly polluted area in North Italy: A population-based study. Heliyon.

[15] Cave M., Appana S., Patel M., Falkner K.C., McClain C.J., Brock G. (2010). Polychlorinated biphenyls, lead, and mercury are associated with liver disease in American adults: NHANES 2003-2004. Environ. Health Perspect..

[16] Kumar J., Lind L., Salihovic S., van Bavel B., Ingelsson E., Lind P.M. (2014). Persistent organic pollutants and liver dysfunction biomarkers in a population-based human sample of men and women. Environ. Res..

[17] Schisterman E.F., Whitcomb B.W., Buck Louis G.M., Louis T.A. (2005). Lipid adjustment in the analysis of environmental contaminants and human health risks. Environ. Health Perspect..

[18] O’Brien K.M., Upson K., Cook N.R., Weinberg C.R. (2016). Environmental Chemicals in Urine and Blood: Improving Methods for Creatinine and Lipid Adjustment. Environ. Health Perspect..

[19] Pierri B., Buonerba C., Coppola A., Pizzolante A., Di Stasio A., Cerino P. (2021). Population-based human biomonitoring in the ‘Land of Fires’ area: Innovations in study design and procedures. Future Sci. OA.

[20] Pierri B., Buonerba C., Pierri A., Pizzolante A., Ferro A., Crispo A., Bollati V., Sanchez T.R., Andreassi M.G., Esposito M. (2022). Exposure study on susceptible people—SPES: An integrative biomonitoring approach. Environ. Int..

[21] Cerino P., Pizzolante A., Buonerba C., Montuori P. (2026). Serum trace metals across environmental-pressure strata in Campania, Italy (SPES): A population-based cross-sectional biomonitoring study. Future Sci. OA.

[22] Buonerba C., Montuori P., Esposito E., Triassi M., Cerino P. (2026). Associations of Serum Trace-Element Concentrations with Selected Blood Biomarkers: A Secondary Cross-Sectional Analysis of the SPES Cohort. Toxics.

[23] Greenland S., Senn S.J., Rothman K.J., Carlin J.B., Poole C., Goodman S.N., Altman D.G. (2016). Statistical tests, P values, confidence intervals, and power: A guide to misinterpretations. Eur. J. Epidemiol..

[24] Walisser J.A., Glover E., Pande K., Liss A.L., Bradfield C.A. (2005). Aryl hydrocarbon receptor-dependent liver development and hepatotoxicity are mediated by different cell types. Proc. Natl. Acad. Sci. USA.

[25] Pierre S., Chevallier A., Teixeira-Clerc F., Ambolet-Camoit A., Bui L.C., Bats A.S., Fournet J.C., Fernandez-Salguero P., Aggerbeck M., Lotersztajn S. (2014). Aryl hydrocarbon receptor-dependent induction of liver fibrosis by dioxin. Toxicol. Sci..

[26] Hakkarainen K., Rantakokko P., Koponen J., Ruokojärvi P., Korkalainen M., Salomaa V., Jula A., Männistö S., Perola M., Lundqvist A. (2023). Persistent organic pollutants associate with liver disease in a Finnish general population sample. Liver Int..

[27] Van Wouwe N., Windal I., Vanderperren H., Eppe G., Xhrouet C., Massart A.-C., Debacker N., Sasse A., Baeyens W., De Pauw E. (2004). Validation of the CALUX bioassay for PCDD/F analyses in human blood plasma and comparison with GC-HRMS. Talanta.

[28] Ayotte P., Dewailly E., Lambert G.H., Perkins S.L., Poon R., Feeley M., Larochelle C., Pereg D. (2005). Biomarker measurements in a coastal fish-eating population environmentally exposed to organochlorines. Environ. Health Perspect..

[29] Li D., Longnecker M.P., Dunson D.B. (2013). Lipid adjustment for chemical exposures: Accounting for concomitant variables. Epidemiology.

[30] Regione Campania, Istituto Zooprofilattico Sperimentale del Mezzogiorno Attività di Medicina Traslazionale per il Biomonitoraggio in Regione Campania. Technical Report; n.d. https://spes.izsmportici.it/wp-content/uploads/2021/06/Relazione-SPES.pdf.

[31] Kwo P.Y., Cohen S.M., Lim J.K. (2017). ACG Clinical Guideline: Evaluation of Abnormal Liver Chemistries. Am. J. Gastroenterol..

[32] Ramaty E., Maor E., Peltz-Sinvani N., Brom A., Grinfeld A., Kivity S., Segev S., Sidi Y., Kessler T., Sela B.A. (2014). Low ALT blood levels predict long-term all-cause mortality among adults. A historical prospective cohort study. Eur. J. Intern. Med..

[33] Visaria A., Pai S., Fayngersh A., Kothari N. (2020). Association between alanine aminotransferase within the normal range and all-cause and cause-specific mortality: A nationwide cohort study. PLoS ONE.

[34] Gallo P., De Vincentis A., Bandinelli S., Ferrucci L., Picardi A., Antonelli Incalzi R., Vespasiani-Gentilucci U. (2021). Combined evaluation of aminotransferases improves risk stratification for overall and cause-specific mortality in older patients. Aging Clin. Exp. Res..

[35] Pettersson J., Hindorf U., Persson P., Bengtsson T., Malmqvist U., Werkström V., Ekelund M. (2008). Muscular exercise can cause highly pathological liver function tests in healthy men. Br. J. Clin. Pharmacol..

[36] Benjamini Y., Hochberg Y. (1995). Controlling the false discovery rate: A practical and powerful approach to multiple testing. J. R. Stat. Soc. Ser. B.

[37] Benjamini Y., Yekutieli D. (2001). The control of the false discovery rate in multiple testing under dependency. Ann. Stat..

[38] Dhooge W., van Larebeke N., Koppen G., Nelen V., Schoeters G., Vlietinck R., Kaufman J.M., Comhaire F., Flemish Environment and Health Study Group (2006). Serum dioxin-like activity is associated with reproductive parameters in young men from the general Flemish population. Environ. Health Perspect..

[39] Clair H.B., Pinkston C.M., Rai S.N., Pavuk M., Dutton N.D., Brock G.N., Prough R.A., Falkner K.C., McClain C.J., Cave M.C. (2018). Liver Disease in a Residential Cohort With Elevated Polychlorinated Biphenyl Exposures. Toxicol. Sci..

[40] Cerino P., De Rosa E., Triassi M., Montuori P., Buonerba C. (2026). Plasma PAH-like AhR Bioactivity, Homocysteine, and Subclinical Vascular and Haematologic Phenotypes in the SPES Biomonitoring Substudy. Int. J. Mol. Sci..

[41] Akins J.R., Waldrep K., Bernert J.T. (1989). The estimation of total serum lipids by a completely enzymatic ‘summation’ method. Clin. Chim. Acta.

[42] Bernert J.T., Turner W.E., Patterson D.G., Needham L.L. (2007). Calculation of serum “total lipid” concentrations for the adjustment of persistent organohalogen toxicant measurements in human samples. Chemosphere.

[43] VanderWeele T.J. (2019). Principles of confounder selection. Eur. J. Epidemiol..

[44] Kenward M.G., Roger J.H. (1997). Small sample inference for fixed effects from restricted maximum likelihood. Biometrics.

[45] Halekoh U., Højsgaard S. (2014). A Kenward–Roger approximation and parametric bootstrap methods for tests in linear mixed models—The R package pbkrtest. J. Stat. Softw..

[46] Holm S. (1979). A simple sequentially rejective multiple test procedure. Scand. J. Stat..

[47] Pustejovsky J.E., Tipton E. (2018). Small-sample methods for cluster-robust variance estimation and hypothesis testing in fixed effects models. J. Bus. Econ. Stat..

[48] von Elm E., Altman D.G., Egger M., Pocock S.J., Gøtzsche P.C., Vandenbroucke J.P., STROBE Initiative (2007). The Strengthening the Reporting of Observational Studies in Epidemiology (STROBE) statement: Guidelines for reporting observational studies. PLoS Med..

[49] Vandenbroucke J.P., von Elm E., Altman D.G., Gøtzsche P.C., Mulrow C.D., Pocock S.J., Poole C., Schlesselman J.J., Egger M., STROBE Initiative (2007). Strengthening the Reporting of Observational Studies in Epidemiology (STROBE): Explanation and elaboration. PLoS Med..

[50] Gallo V., Egger M., McCormack V., Farmer P.B., Ioannidis J.P.A., Kirsch-Volders M., Matullo G., Phillips D.H., Schoket B., Strömberg U. (2011). STrengthening the Reporting of OBservational studies in Epidemiology–Molecular Epidemiology (STROBE-ME): An extension of the STROBE Statement. PLoS Med..

