# Supplementary material for: Plasma DR-CALUX Bioactivity and Concurrent Clinical Biochemistry: A Secondary Cross-Sectional Analysis of SPES
Source: Int J Mol Sci. 2026 Nov 8;27(21):10017. doi: 10.3390/ijms272110017 (PMC13764627; doi:10.3390/ijms272110017)
Supplement: Supplementary file 1 [file ijms-27-10017-s001.zip › ijms-4634178-supplementary.pdf]

Supplementary Material

Plasma DR-CALUX Bioactivity and Concurrent Clinical Biochemistry: A Secondary Cross-Sectional Analysis of SPES

Supplementary Methods, Results, Tables and Figures

This supplement presents the detailed methods, clinical-marker inventory, aggregate results and diagnostics for the SPES DR-CALUX analysis. Tables S1–S15 contain the original analyses; Tables S16–S20 report the selection comparison, joint-benchmark restriction, area-interaction analyses and influence diagnostics added during peer review.

Contents: S1, study population; S2, biospecimens and assay; S3, exposure and lipids; S4, clinical outcomes; S5, covariates and missing data; S6, statistical models; S7, multiplicity; S8, sensitivity, exploratory thyroid and area-interaction analyses; S9, diagnostics and reproducibility. Tables S1–S20 provide the numerical results and descriptive information for the analyses presented in this article. Figures S1–S2 show thyroid spline curves and the global clinical-marker screen. References follow the numbering of the main article.

Supplementary Methods

S1. Study population and analytical scope

This secondary cross-sectional analysis used the 600-participant intensive-biomonitoring subgroup of the Exposure Study on Susceptible People (SPES). The parent programme enrolled 4205 adults in Campania, Italy, during 2016–2017, combining environmental stratification, questionnaires, clinical examination and biospecimen collection. Published reports describe recruitment and implementation [19,20]. The present analysis used unweighted models for the selected subgroup.

The subgroup design allocated 240 places to high-impact areas, 240 to medium-impact areas and 120 to low-impact areas, across sex-by-age strata with age bands of 20–29, 30–39 and 40–49 years. Selection also considered BMI, smoking, alcohol abuse, medication for chronic disease and higher obesity classes [30]. The selection table comprised ten cells of 60 participants, with SPES clusters 20 and 21 combined within one cell; models retained the eleven sampling-cluster codes. The selected roster defined eligibility. Recorded smoking and recent or current medication use were addressed in the covariates and sensitivity analyses described below.

The thyroid and hepatic analysis plan was fixed before the first association run. After completion of the initial analyses, the complete broader-panel specification was finalised before fitting its additional associations. The analysis retained its outcome hierarchy and reported the broader panel as exploratory. The additional selection comparison, joint enzyme-benchmark restriction, pooled area interactions and ALT/AST deletion diagnostics were specified during peer review after the original results were known; they provide supplementary evidence from the same subgroup.

Final laboratory reports supplied quantification status, the BDS/IZSM workbook supplied DR-CALUX values and the clinical subgroup workbook supplied laboratory measurements. Assay and clinical records were linked one-to-one to the selected participant roster. The linked parent dataset supplied alcohol and socioeconomic variables.

The post hoc descriptive comparison used the established 4,188-person analytical reference cohort [21], a subset of the 4,205 original enrolees. It compared the 592 participants in the principal DR-CALUX

analyses with 3,589 reference-cohort participants who were not selected for the original 600-person CALUX subgroup. All 592 principal participants linked to this reference dataset. Seven other CALUX-selected members of the reference cohort were excluded from both comparison groups, leaving 4,181 participants in the comparison. The reference group is therefore restricted to participants outside the original CALUX-selected subgroup. The association-model populations were unchanged.

Both groups used common source fields and variable-specific available and missing denominators. Continuous values are described by medians and interquartile ranges; standardised mean differences (SMDs) use (mean_DR − mean_nonselected)/sqrt[(SD_DR² + SD_nonselected²)/2]. Categorical SMDs use the corresponding pooled Bernoulli variance for each reported level. Missing responses were not coded as negative. Positive finite anthropometric values were retained apart from the previously documented height correction. Recorded impact-area categories were not inferred from sampling-cluster codes. No significance tests, selection weights or comparisons of unmeasured DR-CALUX in nonselected participants were calculated (Table S16). The comparison describes the composition of the actual analytical sample; it cannot separate initial subgroup selection from subsequent analytical exclusions.

Quantifiable DR-CALUX results were available for 594 of the 600 selected participants. Three results were below the limit of quantification (LOQ), one sample was lost during processing and two participants had no assay record. Below-LOQ results were not replaced with assigned numerical values. Two missing alcohol measurements reduced the principal analytical population to 592 participants from 63 municipalities and 11 sampling clusters across three environmental-impact areas.

S2. Biospecimens and DR-CALUX assay

Clinical assessment and blood collection took place at SPES enrolment. Clinical biochemical and haematological measurements formed part of routine testing in laboratories certified for human specimens. Heparinised plasma aliquots were separated, stored at −80 °C and shipped frozen to BioDetection Systems (BDS), Amsterdam, the Netherlands, for DR-CALUX analysis. Clinical lipid concentrations were serum measurements, whereas DR-CALUX used the stored plasma aliquots.

BDS performed the plasma DR-CALUX analyses using its laboratory procedure, identified in the reports as p-bds-051. Plasma extracts underwent acid-silica cleanup. Serial extract dilutions were tested in triplicate for 24 h in stably transfected H4IIe rat hepatoma reporter cells, with plate-specific TCDD calibration relating luminescence to bioassay-equivalent activity. Foundational studies describe the AhR-dependent reporter principle and its application to blood plasma [3,4].

Procedure blanks and reference plasma controls accompanied analysis. Final laboratory-reported values and quantification status determined assay eligibility. The participant was the observational unit, with replicate wells contributing to the laboratory assay measurement.

DR-CALUX measures the activity of material retained through sample preparation in a defined reporter system. The clinical models used this assay-defined bioactivity as the exposure, with the lipid representations described below and the covariates specified in S5. Chemical congener measurements and WHO toxic equivalents address complementary exposure questions. TCDD served as the assay calibrant, expressing the extracted mixture's response relative to that reference under the laboratory conditions.

S3. Exposure definition and alternative lipid models

The primary lipid-normalised exposure was chosen on biological grounds: persistent dioxin-like compounds are lipophilic and partition into circulating lipids. Normalisation expresses recovered activity relative to the circulating lipid compartment. The SPES institutional report already described DR-CALUX bioactivity on a per-lipid basis [30, Section 3.2.3 and Section 4.3.3]. We retained the

archived clinical lipid-component sum as the operational denominator. The clinical component sum and direct gravimetric lipid measurement provide distinct measurement bases, and the individual constituents contributing to assay activity may differ in their partitioning.

Let B denote the positive, quantified, source-reported non-normalised DR-CALUX value. Let TC, TG, PL and FC denote total cholesterol, triglycerides, phospholipids and free cholesterol, respectively, in their recorded mg/dL scales. The primary exposure was the lipid-standardised DR-CALUX index:

$$L_{\mathrm{sum}} = TC + TG + PL, \qquad R_{\mathrm{sum}} = \frac{B}{L_{\mathrm{sum}}/100000}, \qquad X = \log_2(R_{\mathrm{sum}}).$$

The primary model included X without another lipid term. A one-unit increase represents a doubling of the operational bioactivity-to-lipid ratio. The denominator was the sum of the measured lipid components, TC + TG + PL.

The complete Akins estimate was reconstructed as

$$L_{\mathrm{Akins}} = 1.677(TC - FC) + FC + TG + PL = L_{\mathrm{sum}} + 0.677(TC - FC),$$

with $R_{\mathrm{Akins}} = B/(L_{\mathrm{Akins}}/100000)$. Free cholesterol denotes nonesterified cholesterol, not HDL or LDL cholesterol. The full equation retains the $+FC$ term and allows for the fatty-acid contribution to cholesterol esters. Positive TC, TG and PL were required for the lipid-component sum; Akins additionally required finite FC satisfying $0 \leq FC \leq TC$. Akins availability did not determine eligibility for models requiring only the lipid-component sum [41,42].

The ratio was analysed as the operational lipid-standardised exposure index defined above. No participant-specific mass–volume conversion or tissue-dose calibration was applied. A common positive multiplicative change in the numerator shifts its logarithm by a constant and does not change the per-doubling coefficient.

Alternative models assessed lipid handling in four ways: non-normalised bioactivity with estimated total lipids as a separate covariate; non-normalised bioactivity without lipid adjustment; an index standardised using estimated total lipids; and non-normalised bioactivity with the TC + TG + PL component sum as a separate covariate. Bioactivity and lipid terms were $\log_2$-transformed, with freely estimated coefficients in the separate-term models. These were alternative specifications, not successive corrections to an already standardised exposure. The primary coefficient describes a doubling of the bioactivity-to-lipid ratio; separate-term coefficients describe a doubling of bioactivity at fixed modelled lipids. The methodological literature supports different approaches under different assumptions about measurement and confounding [17,18,29].

The lipid-normalised ratio defines the primary exposure contrast. Statistically, $\beta \log_2(B/L)$ equals $\beta \log_2(B) - \beta \log_2(L)$, whereas separate-term models estimate the two coefficients freely. Since circulating lipids can also relate to clinical outcomes, these alternatives assess dependence on the ratio constraint. The direction of a ratio association depends on the relationships among bioactivity, lipids and the outcome. The alternatives complement the biological rationale for normalisation by making the model assumptions explicit. The specified representations were retained irrespective of observed direction or statistical support.

S4. Clinical outcomes and transformations

TSH and FT4 were the two primary thyroid outcomes. FT3 and eight liver-related or protein measurements were secondary outcomes. TSH, ALT, AST, GGT, ALP, total bilirubin, direct bilirubin and pseudocholinesterase were natural-log transformed. FT4, FT3 and albumin retained their identity scales. TSH and FT4 were not mutually adjusted and did not form a composite endpoint.

The exploratory inventory covered all 76 clinical source fields. Fifty-three source fields and one harmonised total-testosterone endpoint yielded 54 analytical outcomes, including the 11 primary and secondary outcomes. Additional domains comprised renal-related chemistry, electrolytes and minerals, glucose, iron-related biochemistry, homocysteine, lactate dehydrogenase, haematology, protein electrophoresis and other endocrine measurements. The inventory contains 77 entries because it includes the harmonised testosterone endpoint and its two source columns. Eligibility required an interpretable quantitative laboratory measurement, correspondence with the enrolment specimen and at least 80% valid observations among the 594 participants with quantifiable DR-CALUX (at least 476 observations). This coverage criterion was not a power calculation. Fewer than ten distinct values prompted review of recording resolution rather than automatic exclusion. Transformations and eligibility were fixed before the additional association models were fitted.

Twenty-three source fields were not tested separately: six lipid measurements, six protein percentages, five differential-cell percentages, the albumin-to-globulin ratio, duplicate albumin, indirect bilirubin, an unpopulated qualitative electrophoresis field and the two testosterone inputs. The latter were combined rather than discarded. Lipid outcomes were outside this ratio-based screen because its denominator components are mathematically coupled to the exposure and related lipid measures require a separately justified exposure model. Absolute differential counts were retained without also testing their percentages. No new inflammatory ratios, eGFR, thyroid-feedback indices or biochemical composite scores were constructed. Blood pressure and other combined vital-sign text fields were outside the laboratory-screen scope.

Additional log-transformed outcomes were urea (reported as azotemia), creatinine, uric acid, cortisol, estradiol, ferritin, FSH, LH, lactate dehydrogenase, homocysteine, progesterone, prolactin, free testosterone and harmonised total testosterone. All other outcomes retained identity scales. Overall, 22 of 54 outcomes were log-transformed and 32 were analysed in their recorded units. Invalid nonpositive values in log outcomes were treated as missing without adding an arbitrary constant. Missingness was handled separately for each outcome.

Total testosterone occupied mutually exclusive columns labelled ng/mL and nmol/L, containing 60 and 540 observations, respectively, with no participant in both or neither. Values in ng/mL were multiplied by 3.47; values already in nmol/L were unchanged. The factor follows the standard ng/dL-to-nmol/L conversion of 0.0347 multiplied by 100.

One albumin field was used consistently, without averaging or substituting duplicate measurements. Total bilirubin equalled direct plus indirect bilirubin in all 600 records. Total and direct bilirubin were retained; indirect bilirubin was excluded as a redundant outcome. Recorded bilirubin values were analysed without substitution or an assumed censoring mechanism.

Recorded absolute protein fractions were analysed directly. TSH was expressed in mIU/L; FT4 and FT3 were expressed in ng/dL and pg/mL. Urea values retained the recorded mg/dL scale. Absolute differential counts used their recorded count scale, listed in Table S3. ALT and AST units of mU/mL are numerically equivalent to U/L. Reference-interval summaries did not change the original continuous outcome models. The separate reviewer-requested joint-benchmark restriction is specified in S8.

S5. Covariates, missing data and analysis populations

The common adjustment set contained age, sex, BMI, recorded current smoking, $\log(1 + \text{alcohol g/day})$, environmental-impact area and a sampling-cluster random intercept. Age and BMI used restricted cubic splines with three knots at their type-7 10th, 50th and 90th percentiles in the 594-participant DR-qualified population, before endpoint-specific exclusions. The age knots were 23, 34 and 46 years; BMI knots were 20.43, 24.44 and 30.17 kg/m². Each spline contributed one linear and one

nonlinear term with linear tails. Knot values are shown rounded; full-precision values calculated from the stated population were reused in every model. Covariates were selected from source definitions and substantive considerations; no stepwise selection, outcome-dependent knot placement or adjustment for another studied outcome was used. The common set provides comparable conditional associations but is not asserted to eliminate confounding for every phenotype [43].

The principal models retained both recorded current smokers among the 594 participants with quantifiable DR-CALUX. Alcohol was recorded in g/day; missing values were not recoded to zero. Two missing alcohol values yielded 592 complete cases for all 11 primary and secondary outcomes. Across the 54-marker panel, 39 outcomes had 592 observations, 14 haematological outcomes had 563 and mean platelet volume had 562. Participants missing the blood-count panel were concentrated in sampling clusters 20 and 21.

Models used observed, outcome-specific complete cases, with sample sizes accompanying each estimate. The common covariate definitions were retained in pooled endocrine models, which assumed a shared exposure slope across sexes; interactions were examined for TSH and FT4. The broader SPES questionnaires recorded self-reported histories, including current oral-contraceptive and menopausal-hormone use. These items remained outside the adjustment set. Their limited clinical detail and alignment with physiological status at blood collection restrict the resolution of additional endocrine-specific adjustment. Reproductive state, menstrual phase, individual collection timing and hormone-specific treatment therefore remain sources of residual variation.

The administered clinical case-report form combined reported history with investigator assessment. Its medication item asked about use during the previous six months or currently: 126 DR-qualified participants answered yes, 466 no and two had unknown responses. The medication-free restriction required an explicit no and retained 464 participants after core completeness requirements. Drug names were available for reported users but were not adjudicated into therapeutic classes for this analysis. A generic medication response was not treated as thyroid treatment. The individual deprivation score combined education and household resources and was distinct from an area-level deprivation measure; its sensitivity coefficient was specified per ten score units.

Structured questionnaire items concerning physician-diagnosed high cholesterol/triglycerides and diabetes, selected current treatments and a broad occupational-exposure indicator were collected for wider population characterisation and remained outside the principal adjustment set. The recorded histories offered limited detail on disease severity, treatment class, dose and adherence, and specific occupational agents or exposure intensity. Comprehensive chronic-liver-disease characterisation remained limited. The liver-stones item retained its specific meaning; disease and treatment classifications in this analysis used the defined structured items. The reported models retained the original adjustment strategy and targeted sensitivities. Further clinical models using these historical records would require additional post hoc definitions and analytical choices.

S6. Model specification, inference and analytical support

For outcome Y_{ij} in participant i and sampling cluster j, the primary mean model was

$$g(Y_{ij}) = \alpha + \beta X_{ij} + f_A(\mathrm{age}_{ij}) + f_B(\mathrm{BMI}_{ij}) + \boldsymbol{\theta}^T \mathbf{Z}_{ij} + u_j + \epsilon_{ij},$$

where g was the specified identity or natural-log transformation. The vector $\mathbf{Z}_{ij}$ contains sex, recorded current smoking, log(1 + alcohol g/day) and environmental-impact-area indicators; $\boldsymbol{\theta}$ contains their coefficients. The functions f_A and f_B are the fixed age and BMI spline bases described in S5. Cluster intercepts and participant residuals were modelled as independent normal random quantities with separate variances, and cluster intercepts were assumed independent of included predictors.

Municipality, sampling cluster and impact area were nested in the study design. Area fixed effects and all nested cluster indicators were not simultaneously fitted as unrestricted fixed effects.

Mixed models used restricted maximum likelihood. Kenward–Roger adjusted fixed-effect covariance and contrast-specific denominator degrees of freedom supplied both the two-sided exposure test and pointwise 95% confidence interval. For identity outcomes, β is the adjusted difference in source units per exposure doubling. For log outcomes, percentage differences in conditional geometric means and interval limits were calculated as $100[\exp(\beta) - 1]$, applying the same transformation separately to both coefficient limits. The intervals were not simultaneous or adjusted for selection [44,45].

The design matrix had to have full rank, the exposure had to vary and the number of observations had to exceed the number of fixed-effect coefficients by more than two. Mixed models required at least two represented sampling clusters, fewer clusters than observations, successful convergence and a finite estimable contrast with positive finite standard error and denominator degrees of freedom. The fixed optimizer was bobyqa with at most 200,000 function evaluations. A nonconstant aliased term was not dropped. A singular random-intercept fit, assessed at tolerance 10^{-4}, was retained only when the otherwise specified model converged and its contrast remained supported; singularity was flagged rather than triggering an ordinary-least-squares substitution. Failed models retained an explicit unavailable status.

The geographic sensitivity replaced both impact-area fixed effects and the random intercept with sampling-cluster fixed effects and used municipality-clustered CR2 covariance with exposure-specific Satterthwaite inference. Its exposure coefficient uses within-sampling-cluster variation after adjustment for the other covariates. CR2 permits residual dependence within municipalities but assumes independence between municipalities; it does not retain a shared random covariance across different municipalities in the same sampling cluster. It therefore altered both the mean and covariance assumptions. In addition to rank and estimability requirements, this comparison required at least 50 observations, at least 20 represented municipalities and finite exposure-specific degrees of freedom of at least 10. These were prespecified pragmatic support rules, not universal CR2 validity thresholds [47].

S7. Outcome hierarchy and multiplicity

The primary and secondary analyses used four outcome families. Holm adjustment controlled the familywise error rate across TSH and FT4 at 0.05; each endpoint could support its own conclusion, without requiring both to meet the threshold. FT3 was a secondary singleton. Benjamini–Hochberg (BH) adjustment was applied separately to the six enzyme and bilirubin outcomes and the two protein-related outcomes, using an adjusted-p-value threshold of 0.05. These family-specific procedures did not provide one study-wide error guarantee across all 11 outcomes or the exploratory thyroid tests [36,46].

All 54 clinical outcomes, including the 11 primary and secondary outcomes, also formed one exploratory family. Its global criterion was a Benjamini–Yekutieli (BY) adjusted p value of at most 0.05. BY controls the false discovery rate under arbitrary dependence, conditional on valid individual tests. BH-adjusted values were reported as a less conservative comparison, not as an alternative route to satisfy the BY criterion [36,37].

The original principal exposure models supplied the thyroid, secondary-family and global-panel tests described above. Planned outcomes that could not be estimated retained their place in the adjustment family using $p = 1$ internally, while their displayed results remained unavailable. All outcomes were reported regardless of effect direction or statistical support. The later area-interaction family is defined separately in S8; the additional restriction and covariate sensitivities supplied estimates and pointwise intervals, not new inferential families.

S8. Sensitivity analyses and exploratory thyroid models

In the original analysis, thirteen sensitivity variants were applied to the 11 primary and secondary outcomes, with the scopes shown below. Each comparison started from the corresponding principal complete-case population unless explicitly defined otherwise. A primary model was refitted on the same participants when a restriction reduced the sensitivity sample.

Variant	Specification	Outcome scope
Design-context adjustment	Retain age, sex, impact area and cluster random intercept; omit BMI, smoking and alcohol on the same primary sample	11 primary and secondary
Bioactivity adjusted for estimated total lipids	Log_2 non-normalised bioactivity plus a separate log_2 estimated-total-lipid term	11 primary and secondary; all 54 outcomes
Bioactivity without lipid adjustment	Log_2 non-normalised bioactivity without a lipid term	11 primary and secondary
Index using estimated total lipids	Log_2 Akins-normalised ratio without a separate lipid term	11 primary and secondary
Bioactivity adjusted for lipid-component sum	Log_2 non-normalised bioactivity plus a separate log_2 lipid-component-sum term	11 primary and secondary; all 54 outcomes
Alternative geographic adjustment	Sampling-cluster fixed effects, no separate impact-area term, municipality CR2 inference	11 primary and secondary; all 54 outcomes
BMI omission	Remove both BMI spline terms	TSH and FT4
Current-smoker exclusion	Restrict to recorded nonsmokers and remove the smoking term	TSH and FT4
Upper-tail exclusion	Exclude primary-index values strictly above the frozen 99th percentile	TSH and FT4
Alcohol omission, matched	Remove alcohol on the primary complete-case sample	11 primary and secondary
Alcohol omission, expanded	Remove alcohol and use all otherwise eligible DR-qualified participants	11 primary and secondary
Deprivation extension	Add the individual deprivation score as one linear term	11 primary and secondary
Medication-free restriction	Require an explicit no to the CRF medication item	11 primary and secondary

Sensitivity analyses reported estimates, pointwise confidence intervals and sample sizes, without additional p-value families. All 54 outcomes were examined using bioactivity adjusted for estimated total lipids, bioactivity adjusted for the lipid-component sum, and alternative geographic adjustment. These 162 estimates include the corresponding 33 estimates for the 11 primary and secondary outcomes; the duplicated entries do not constitute independent corroboration. Other sensitivity variants retained their specified outcome scopes.

The upper-tail threshold was 94.30 (shown rounded) on the primary-index scale, the type-7 99th percentile in the 594-person qualified population. Values tied at the threshold remained; source values were not winsorised. The executed variants and their outcome scopes are listed in the table above.

Exposure nonlinearity and exposure-by-sex interaction were confined to TSH and FT4. Three-knot restricted cubic splines of log_2 primary index used knots 4.8173, 5.3997 and 6.0305 (shown rounded; full-precision values were calculated from the stated population). The two-parameter global exposure tests formed one BH family of two, and the one-parameter nonlinear tests a second family of two. Both used the corresponding Kenward–Roger adjusted F reference. The two pooled linear exposure-by-sex interaction tests formed a third BH family of two. Female and male slopes were estimated as contrasts of the pooled model, rather than by comparing subgroup significance labels. The primary linear model remained unchanged by these findings. Spline curves used the common qualified-population median as zero contrast and were displayed between its first and 99th exposure percentiles with pointwise confidence intervals.

Reviewer-requested aminotransferase sensitivity. Both original enzyme models were refitted after requiring 0 < ALT ≤ 59 U/L and 0 < AST ≤ 40 U/L (n = 569). The upper limits were common benchmarks from an example report for an adult man, not verified sex-specific limits for every participant. Nineteen participants exceeded the ALT upper limit, 18 the AST upper limit and 14 both; the joint restriction excluded 23. Original covariates, transformations and full-precision spline knots were retained. Estimates and pointwise intervals are reported alongside the original models in Table S17. Conditioning on two outcomes changes the target population and can change the slope; this is not a correction for confounding or a comprehensive exclusion of liver disease.

Reviewer-requested area heterogeneity. A pooled model for each of ALT and AST included the main effects of $\log_2$ exposure and the three-level area factor, their two interaction coefficients, all other original covariates and the sampling-cluster random intercept. The two interaction coefficients were jointly tested using the adjusted Kenward–Roger F test with two numerator degrees of freedom; BH adjustment covered the ALT and AST omnibus tests only. All area-specific slopes were linear contrasts using the full adjusted covariance matrix and contrast-specific denominator degrees of freedom, with pointwise intervals. No separate area model was chosen by its significance. Table S18 reports the tests, slopes and area-specific participant, municipality, cluster and $\log_2$ exposure support.

S9. Diagnostics and reproducibility

Residual-versus-fitted and normal quantile–quantile plots, standardised conditional residuals, convergence and singularity records accompanied every principal outcome model. Counts of absolute standardised residuals exceeding three were reported as diagnostics; they did not trigger exclusions. For TSH and FT4, the specified model was additionally refitted after deleting each participant and each municipality in turn. Influence was summarised as change in the exposure coefficient and change divided by the full-model Kenward–Roger standard error. These original refits supplied neither new inferential tests nor exclusion decisions and were confined to TSH and FT4 (Table S12).

During peer review, the same original ALT and AST specifications were additionally refitted after deleting each of 592 participants, 63 municipalities and 11 sampling clusters in turn: 1,332 refits in total. No new Kenward–Roger covariance was calculated for individual deletion fits. Summaries include availability, exposure-effect ranges, maximum coefficient change divided by the full-model Kenward–Roger standard error, and boundary-fit counts (Table S20). This scaled coefficient shift is descriptive, not exact deletion DFBETAS. No residual or influence measure caused exclusion from the original models. No thyroid sampling-cluster-deletion analysis was performed.

Table S19 reports random-intercept and residual variances, intraclass correlations and exposure-specific Kenward–Roger degrees of freedom for the four original models. Intraclass correlation is random-intercept variance divided by random-intercept plus residual variance; Table S13 gives the residual summaries. With eleven sampling clusters, between-cluster variance and inference remain sensitive to covariance assumptions. The alternative CR2 analysis assumes independence between municipalities. The deletion summaries describe coefficient stability, while the limited cluster support and residual-distribution departures remain qualifications to interval calibration.

Association analyses used R version 4.6.1 on Windows 11 x64 (Microsoft Corporation, Redmond, WA, USA), with lme4 2.0-6, pbkrtest 0.5.5 and clubSandwich 0.7.0. Aggregate tables were generated from unrounded estimates, with rounding restricted to presentation. Data-free analytical code and supporting aggregate files are provided separately for editorial and peer review in Non-published Material.zip; participant-level inputs are not included. The descriptive comparison was calculated in Python (Python Software Foundation, Beaverton, OR, USA) from the linked source records using the group and summary definitions in S1.

Supplementary Results

All 54 principal estimates and 162 broad sensitivity estimates were obtained. Eleven principal and 23 sensitivity mixed models had boundary random-intercept variance and were retained as converged fits with estimable contrasts. All global BY-adjusted p values exceeded 0.05; ALT and AST met the BH comparison criterion. Additional results comprise 116 sensitivity estimates across 13 variants, 15 matched principal refits, six exploratory thyroid tests, four sex-specific contrasts and 1310 participant- or municipality-deletion refits.

Tables report their outcome-specific denominators and multiplicity families. Confidence intervals are pointwise. All eligible clinical markers are presented, including associations that did not meet an adjusted significance threshold.

Tables S16–S20 report the additional analyses presented in this article. The selection comparison uses the 4,188-person reference cohort; the association analyses use the original 592-person sample or the joint-benchmark subset of 569. The benchmark-restricted and interaction models were estimable. All 1,332 ALT/AST deletion refits were available and retained inverse coefficients; one AST municipality-deletion fit and one AST sampling-cluster-deletion fit had a boundary variance.

Table S1. Participant flow and analytical denominators.

Stage	Number	Explanation
Parent programme enrolled	4205	Published SPES programme
Selected CALUX substudy	600	Implemented selection, no population weighting
Assay-report records	598	Two selected participants absent from assay roster
Quantified DR-CALUX	594	Three below LOQ; one processing loss among the 598
Core complete cases	592	Two required alcohol values missing
11 primary and secondary endpoints	592 each	Same endpoint-specific complete population
39 non-haematological outcomes	592 each	Non-CBC portion of eligible panel
14 haematological outcomes	563 each	Outcome-specific missing blood-count measurements
Mean platelet volume	562	Outcome-specific missing laboratory measurements
Sampling support	11 clusters; 63 municipalities	Three environmental-impact areas

LOQ, limit of quantification; CBC, complete blood count. Losses apply to their stated stage, not as additional exclusions from the 600-person sample.

Table S2. Exposure components and participant characteristics.

Characteristic	Quantified DR (n = 594)	Principal models (n = 592)
Age (years)	34 [27, 42]	34 [27, 42]
BMI (kg/m²)	24.44 [22.41, 27.34]	24.44 [22.43, 27.34]
Alcohol (g/day)	0.15 [0.15, 6.07]; n=592	0.15 [0.15, 6.07]
Non-normalised DR-CALUX (source scale)	0.227 [0.183, 0.291]	0.227 [0.183, 0.290]
Lipid-standardised DR-CALUX index (operational scale)	42.22 [33.54, 53.52]	42.22 [33.64, 53.39]
Total cholesterol (mg/dL)	185.0 [163.0, 210.0]	185.0 [163.0, 210.0]
Free cholesterol (mg/dL)	34.4 [28.5, 41.6]	34.4 [28.5, 41.6]
Triglycerides (mg/dL)	85.0 [68.0, 114.8]	85.0 [68.0, 115.0]
Phospholipids (mg/dL)	254.0 [229.0, 282.0]	254.0 [229.0, 282.0]
Lipid-component sum (mg/dL)	532.5 [475.0, 602.0]	532.5 [474.8, 602.2]
Akins estimated total lipids (mg/dL)	627.7 [566.3, 717.2]	627.7 [566.4, 717.0]
Women	299 (50.3%)	298 (50.3%)
Men	295 (49.7%)	294 (49.7%)
Recorded current smoking	2 (0.3%)	2 (0.3%)
Low-impact area	120 (20.2%)	120 (20.3%)
Medium-impact area	238 (40.1%)	237 (40.0%)
High-impact area	236 (39.7%)	235 (39.7%)
Recent/current medication: yes	126 (21.2%)	126 (21.3%)
Recent/current medication: no	466 (78.5%)	464 (78.4%)
Recent/current medication: unknown	2 (0.3%)	2 (0.3%)

Continuous values are median [IQR]; categorical values are n (%), using the column denominator. BMI, body mass index; DR-CALUX, dioxin-responsive chemically activated luciferase gene expression. Bioactivity and the lipid-standardised DR-CALUX index are reported on their source numerical scales; S3 defines the ratio. Alcohol is observed for 592 participants in both columns. All primary and secondary endpoint models use the 592-person population.

Table S3. Complete clinical-field inventory and eligibility decisions.

Field / endpoint	Column	Reported unit	Transform	Valid /594	Core n	Use	Reason
Calcium	W	mg/dL	identity	594	592	modelled	Recorded total serum calcium; no albumin-corrected value constructed
Chloride	X	mmol/L	identity	594	592	modelled	Recorded serum concentration
Phosphorus	AK	mg/dL	identity	594	592	modelled	Source serum phosphorus convention
Potassium	BM	mmol/L	identity	594	592	modelled	Recorded serum concentration
Sodium	BU	mmol./L	identity	594	592	modelled	Recorded serum concentration
Glucose	AR	mg/dL	identity	594	592	modelled	Recorded serum glucose
Basophils (%)	N	%	identity	565	563	excluded	Percentage counterpart of retained absolute basophil count
Basophils (absolute count)	O	10e3/µL	identity	565	563	modelled	Absolute count on the recorded numerical scale
Eosinophils (%)	AF	%	identity	565	563	excluded	Percentage counterpart of retained absolute eosinophil count
Eosinophils (absolute count)	AE	10e3/µL	identity	565	563	modelled	Two recorded zeros retained as counts; no pseudocount or log exclusion
Haematocrit	AC	%	identity	565	563	modelled	Distinct CBC quantity; correlated with Hb and RBC
Haemoglobin	AD	g/dL	identity	565	563	modelled	Distinct CBC quantity; correlated with haematocrit and RBC
Lymphocytes (%)	BA	%	identity	565	563	excluded	Percentage counterpart of retained absolute lymphocyte count
Lymphocytes (absolute count)	BB	10e3/µL	identity	565	563	modelled	Absolute count on the recorded numerical scale
Mean corpuscular haemoglobin (MCH)	BC	pg	identity	565	563	modelled	Reported CBC index; not an independent replication of other red-cell markers
Mean corpuscular haemoglobin concentration (MCHC)	BD	g/dL	identity	565	563	modelled	Reported CBC index; not an independent replication of other red-cell markers
Mean corpuscular volume (MCV)	BE	fL	identity	565	563	modelled	Reported CBC index
Mean platelet volume (MPV)	BH	fL	identity	564	562	modelled	One recorded zero treated as missing for MPV only
Monocytes (%)	BF	%	identity	565	563	excluded	Percentage counterpart of retained absolute monocyte count
Monocytes (absolute count)	BG	10e3/µL	identity	565	563	modelled	Absolute count on the recorded numerical scale
Neutrophils (%)	BJ	%	identity	565	563	excluded	Percentage counterpart of retained absolute neutrophil count
Neutrophils (absolute count)	BI	10e3/µL	identity	565	563	modelled	Absolute count on the recorded numerical scale

Field / endpoint	Column	Reported unit	Transform	Valid /594	Core n	Use	Reason
Platelets	BL	/uL	identity	565	563	modelled	Platelet count; source /uL scale retained
Red blood cells	AT	/uL	identity	565	563	modelled	Red-cell count; source /uL scale retained
Red-cell distribution width (RDW)	BS	%	identity	565	563	modelled	Reported CBC index
White blood cells	AS	/uL	identity	565	563	modelled	Total white-cell count; source /uL scale retained
Alanine aminotransferase (ALT)	AV	mU/ml	ln	594	592	modelled	Secondary outcome
Alkaline phosphatase (ALP)	AI	UI/l	ln	594	592	modelled	Secondary outcome
Aspartate aminotransferase (AST)	AU	mU/ml	ln	594	592	modelled	Secondary outcome
Direct bilirubin	V	mg/dL	ln	594	592	modelled	Secondary outcome; recorded values retained
Gamma-glutamyltransferase (GGT)	AQ	U/l	ln	594	592	modelled	Secondary outcome
Indirect bilirubin	U	mg/dL	ln	594	592	excluded	Exact arithmetic redundancy with retained total and direct bilirubin
Total bilirubin	T	mg/dL	ln	594	592	modelled	Secondary outcome; exact total equals direct plus indirect in all 600
Ferritin	AH	ng/ml	ln	594	592	modelled	Recorded ferritin
Serum iron	BT	mcg/dL	identity	594	592	modelled	Serum iron chemistry; distinct from ICP-MS blood iron exposure
Transferrin	BY	mg/dL	identity	594	592	modelled	Recorded serum concentration
Free cholesterol	Y	mg/dL	identity	594	592	excluded	Lipid-domain outcome excluded from lipid-ratio exposure screen
HDL cholesterol	AW	mg/dL	identity	594	592	excluded	Lipid-domain outcome excluded from lipid-ratio exposure screen
LDL cholesterol	AY	mg/dL	identity	594	592	excluded	Lipid-domain outcome excluded from lipid-ratio exposure screen
Phospholipids	AJ	mg/dL	identity	594	592	excluded	Component of exposure denominator; excluded from lipid-ratio exposure screen
Total cholesterol	Z	mg/dL	identity	594	592	excluded	Component of exposure denominator; excluded from lipid-ratio exposure screen
Triglycerides	BZ	mg/dL	identity	594	592	excluded	Component of exposure denominator; excluded from lipid-ratio exposure screen

Field / endpoint	Column	Reported unit	Transform	Valid /594	Core n	Use	Reason
Homocysteine	BK	micromol/L	ln	594	592	modelled	Recorded concentration
Cortisol	AA	mcg/dL	ln	594	592	modelled	Pooled sex-adjusted screen; no collection-time or stress adjustment
Estradiol	AG	pg/mL	ln	594	592	modelled	Pooled sex-adjusted screen; menstrual phase and menopausal status not controlled
Follicle-stimulating hormone (FSH)	AL	mIU/L	ln	594	592	modelled	Pooled sex-adjusted screen; menstrual phase and menopausal status not controlled
Free testosterone	BX	pg/mL	ln	594	592	modelled	Single source unit; pooled sex-adjusted screen
Luteinising hormone (LH)	AZ	mIU/L	ln	594	592	modelled	Pooled sex-adjusted screen; menstrual phase and menopausal status not controlled
Progesterone	BN	ng/ml	ln	594	592	modelled	Pooled sex-adjusted screen; menstrual phase and menopausal status not controlled
Prolactin	BO	ng/ml	ln	594	592	modelled	Pooled sex-adjusted screen; medication classes and sampling stress unadjusted
Total testosterone (harmonised)	BV+BW	nmol/L	ln	594	592	modelled	One endpoint from mutually exclusive source columns: BV ng/mL multiplied by 3.47; BW nmol/L retained
Total testosterone (ng/mL column)	BV	ng/ml	ln	60	60	Harmonization input	Input to one harmonised total_testosterone endpoint; multiply explicitly labelled ng/mL values by 3.47; not tested separately
Total testosterone (nmol/L column)	BW	nmol/L	ln	534	532	Harmonization input	Input to one harmonised total_testosterone endpoint; retain explicitly labelled nmol/L values; not tested separately
Lactate dehydrogenase (LDH)	AX	U/l	ln	594	592	modelled	Lactate dehydrogenase is nonspecific; no organ-specific interpretation
Albumin (%)	F	%	identity	594	592	excluded	Percentage counterpart of retained absolute albumin
Albumin-to-globulin ratio	E	ratio	identity	594	592	excluded	Derived ratio of albumin and globulins; component-based redundancy
Alpha-1 globulin	J	g/dL	identity	594	592	modelled	Absolute electrophoretic fraction; correlated with total protein and other fractions
Alpha-1 globulin (%)	I	%	identity	594	592	excluded	Percentage counterpart of retained absolute alpha-1 globulin
Alpha-2 globulin	K	g/dL	identity	594	592	modelled	Absolute electrophoretic fraction; correlated with total protein and other fractions

Field / endpoint	Column	Reported unit	Transform	Valid /594	Core n	Use	Reason
Alpha-2 globulin (%)	L	%	identity	594	592	excluded	Percentage counterpart of retained absolute alpha-2 globulin
Beta-1 globulin	P	g/dL	identity	594	592	modelled	Absolute electrophoretic fraction; correlated with total protein and other fractions
Beta-1 globulin (%)	Q	%	identity	594	592	excluded	Percentage counterpart of retained absolute beta-1 globulin
Beta-2 globulin	S	g/dL	identity	594	592	modelled	Absolute electrophoretic fraction; correlated with total protein and other fractions
Beta-2 globulin (%)	R	%	identity	594	592	excluded	Percentage counterpart of retained absolute beta-2 globulin
Electrophoresis description	BR	—	identity	0	0	excluded	No recorded values in all 600 participants
Gamma globulin	AP	g/dL	identity	594	592	modelled	Absolute electrophoretic fraction; correlated with total protein and other fractions
Gamma globulin (%)	AO	%	identity	594	592	excluded	Percentage counterpart of retained absolute gamma globulin
Total protein	BP	g/dL	identity	594	592	modelled	Total concentration; electrophoretic fractions not independent outcomes
Albumin	G	g/dL	identity	594	592	modelled	Column G used consistently
Albumin (duplicate column H)	H	g/dL	identity	594	592	excluded	Duplicate field; column G used consistently
Pseudocholinesterase	BQ	U/l	ln	594	592	modelled	Secondary outcome
Urea	M	mg/dL	ln	594	592	modelled	Urea; source field named azotemia
Creatinine	AB	mg/dL	ln	594	592	modelled	Recorded creatinine; no derived eGFR or diagnostic cutoffs
Uric acid	CB	mg/dL	ln	594	592	modelled	Recorded serum concentration
Free thyroxine (FT4)	AN	ng/dL	identity	594	592	modelled	Primary thyroid outcome
Free triiodothyronine (FT3)	AM	pg/mL	identity	594	592	modelled	Secondary thyroid outcome
Thyroid-stimulating hormone (TSH)	CA	mIU/L	ln	594	592	modelled	Primary thyroid outcome

Seventy-seven entries comprise the 76 source clinical fields plus one harmonised total-testosterone endpoint. Fifty-four outcomes were analysed. Of 23 fields not modelled individually, two are inputs to harmonised testosterone; they were combined, not discarded. “ln” is natural log; “identity” retains the numerical source scale. Units follow the study documentation, with original count notation retained for differential counts. Source columns refer to Dataset_Biochimica; harmonisation is described in S4. Rows are grouped by domain for readability; eligibility was fixed before added associations.

Table S4. Clinical-marker distributions and model populations.

Outcome	Reported unit	Observed /594	Model n	Median [IQR]	Range	Transform
Calcium	mg/dL	594	592	9.100 [8.800, 9.400]	8.000–10.200	identity
Chloride	mmol/L	594	592	104.000 [102.000, 106.000]	97.000–110.000	identity
Phosphorus	mg/dL	594	592	3.800 [3.400, 4.275]	2.000–6.400	identity
Potassium	mmol/L	594	592	4.600 [4.200, 4.800]	3.500–5.600	identity
Sodium	mmol./L	594	592	140.000 [138.000, 141.000]	134.000–145.000	identity
Glucose	mg/dL	594	592	77.000 [72.000, 84.000]	65.000–120.000	identity
Basophils (absolute count)	10e3/µL	565	563	0.070 [0.050, 0.090]	0.010–0.290	identity
Eosinophils (absolute count)	10e3/µL	565	563	0.120 [0.080, 0.200]	0.000–0.830	identity
Haematocrit	%	565	563	41.100 [38.040, 44.040]	26.990–58.120	identity
Haemoglobin	g/dL	565	563	13.990 [12.890, 15.040]	8.980–20.010	identity
Lymphocytes (absolute count)	10e3/µL	565	563	2.050 [1.700, 2.500]	0.710–4.820	identity
Mean corpuscular haemoglobin (MCH)	pg	565	563	29.190 [28.210, 30.000]	17.780–33.900	identity
Mean corpuscular haemoglobin concentration (MCHC)	g/dL	565	563	34.190 [33.420, 34.830]	26.940–36.800	identity
Mean corpuscular volume (MCV)	fL	565	563	85.020 [82.400, 87.280]	57.360–104.900	identity
Mean platelet volume (MPV)	fL	564	562	7.450 [6.650, 8.480]	4.890–13.610	identity
Monocytes (absolute count)	10e3/µL	565	563	0.450 [0.370, 0.560]	0.170–1.310	identity
Neutrophils (absolute count)	10e3/µL	565	563	3.360 [2.660, 4.160]	0.700–9.200	identity
Platelets	/uL	565	563	224200.000 [197200.000, 259900.000]	92430.000–451600.000	identity
Red blood cells	/uL	565	563	4841000.000 [4511000.000, 5208000.000]	3682000.000–7120000.000	identity
Red-cell distribution width (RDW)	%	565	563	12.060 [11.650, 12.600]	10.450–18.130	identity
White blood cells	/uL	565	563	6196.000 [5243.000, 7381.000]	2791.000–13320.000	identity
ALT	mU/ml	594	592	35.000 [30.000, 40.000]	10.000–138.000	ln

Outcome	Reported unit	Observed /594	Model n	Median [IQR]	Range	Transform
ALP	UI/l	594	592	60.000 [48.000, 72.000]	24.000–154.000	ln
AST	mU/ml	594	592	23.000 [19.000, 28.000]	10.000–242.000	ln
Direct bilirubin	mg/dL	594	592	0.300 [0.200, 0.400]	0.100–1.100	ln
GGT	U/l	594	592	19.000 [14.000, 28.000]	9.000–216.000	ln
Total bilirubin	mg/dL	594	592	0.700 [0.500, 0.900]	0.200–3.400	ln
Ferritin	ng/ml	594	592	59.500 [24.800, 152.700]	2.400–1708.300	ln
Serum iron	mcg/dL	594	592	86.500 [67.000, 110.000]	17.000–247.000	identity
Transferrin	mg/dL	594	592	260.000 [235.000, 291.000]	143.000–475.000	identity
Homocysteine	micromol/L	594	592	8.100 [7.200, 9.300]	4.800–17.000	ln
Cortisol	mcg/dL	594	592	8.200 [7.100, 10.000]	3.000–29.300	ln
Estradiol	pg/mL	594	592	19.745 [17.100, 22.500]	6.800–201.000	ln
Follicle-stimulating hormone (FSH)	mIU/L	594	592	4.500 [2.900, 6.700]	0.050–101.700	ln
Free testosterone	pg/mL	594	592	6.450 [0.345, 11.900]	0.020–28.000	ln
Luteinising hormone (LH)	mIU/L	594	592	4.250 [2.930, 6.285]	0.030–62.540	ln
Progesterone	ng/ml	594	592	0.340 [0.240, 0.508]	0.100–38.600	ln
Prolactin	ng/ml	594	592	13.730 [9.822, 19.587]	3.560–127.360	ln
Total testosterone (harmonised)	nmol/L	594	592	2.863 [1.120, 17.037]	0.278–37.690	ln
Lactate dehydrogenase (LDH)	U/l	594	592	154.000 [139.000, 174.000]	118.000–297.000	ln
Alpha-1 globulin	g/dL	594	592	0.270 [0.250, 0.300]	0.170–0.450	identity
Alpha-2 globulin	g/dL	594	592	0.650 [0.590, 0.710]	0.140–1.060	identity
Beta-1 globulin	g/dL	594	592	0.470 [0.440, 0.510]	0.320–0.720	identity
Beta-2 globulin	g/dL	594	592	0.290 [0.250, 0.320]	0.150–0.750	identity
Gamma globulin	g/dL	594	592	1.140 [1.000, 1.290]	0.620–1.860	identity
Total protein	g/dL	594	592	7.200 [6.900, 7.500]	6.000–8.300	identity

Outcome	Reported unit	Observed /594	Model n	Median [IQR]	Range	Transform
Albumin	g/dL	594	592	4.300 [4.140, 4.518]	3.480–5.460	identity
Pseudocholinesterase	U/l	594	592	10196.000 [8835.500, 10999.000]	3716.000–13208.000	ln
Urea	mg/dL	594	592	34.000 [28.000, 39.000]	13.000–85.000	ln
Creatinine	mg/dL	594	592	0.800 [0.700, 0.900]	0.500–2.500	ln
Uric acid	mg/dL	594	592	4.700 [3.900, 5.700]	1.600–9.600	ln
FT4	ng/dL	594	592	1.050 [0.980, 1.140]	0.700–1.480	identity
FT3	pg/mL	594	592	3.410 [3.150, 3.620]	1.710–3.870	identity
TSH	mIU/L	594	592	1.775 [1.292, 2.430]	0.100–12.780	ln

Descriptive statistics use the 594 participants with quantifiable DR-CALUX and available outcome measurements. Model n additionally requires complete covariate data. Units follow Table S3; mU/mL and U/L are numerically equivalent. The complete aggregate results are presented in the tables of this supplement.

Table S5. Primary and secondary associations for the 11 prioritised outcomes.

Outcome	Scale	Difference (95% CI)	Raw p	Adjusted p	Family
TSH	%	−7.23 (−14.96, 1.21)	0.0911	0.182	Holm, 2
FT4	ng/dL	−0.0047 (−0.0233, 0.0139)	0.621	0.621	Holm, 2
FT3	pg/mL	0.0291 (−0.0337, 0.0919)	0.363	0.363	Singleton
ALT	%	−9.33 (−14.22, −4.17)	0.00054	0.00195	BH, 6
AST	%	−8.01 (−12.31, −3.50)	0.00065	0.00195	BH, 6
GGT	%	−7.27 (−13.97, −0.04)	0.0488	0.0586	BH, 6
ALP	%	−5.22 (−9.46, −0.78)	0.0217	0.0325	BH, 6
Total bilirubin	%	5.25 (−2.44, 13.56)	0.186	0.186	BH, 6
Direct bilirubin	%	9.73 (1.85, 18.22)	0.0148	0.0295	BH, 6
Albumin	g/dL	−0.0434 (−0.0863, −0.0004)	0.0477	0.0743	BH, 2
Pseudocholinesterase	%	−2.27 (−4.71, 0.23)	0.0743	0.0743	BH, 2

Effects are per doubling of the lipid-standardised DR-CALUX index. All models: n = 592; 11 sampling clusters; 63 municipalities. Adjustment: age and BMI restricted cubic splines, sex, current smoking, log(1 + alcohol), environmental-impact area, and sampling-cluster random intercept. REML with Kenward–Roger inference. Percentages are differences in conditional geometric means; other effects are native-unit mean differences. Intervals are pointwise. BH, Benjamini–Hochberg; CI, confidence interval; REML, restricted maximum likelihood. FT3 is a secondary singleton without multiplicity adjustment. TSH and direct bilirubin had boundary random-intercept variance and were retained. ALT, alanine aminotransferase; AST, aspartate aminotransferase; ALP, alkaline phosphatase; GGT, gamma-glutamyltransferase; FT3/FT4, free triiodothyronine/thyroxine; TSH, thyroid-stimulating hormone.

Table S6. All 54 exploratory associations and both global corrections.

Outcome	Scale	n	Difference (95% CI)	Raw p	BY adjusted p	BH comparison	State
Calcium	mg/dL	592	−0.014 (−0.072, 0.045)	0.651	1.000	0.760	Regular
Chloride	mmol/L	592	−0.138 (−0.527, 0.251)	0.486	1.000	0.656	Regular
Phosphorus	mg/dL	592	−0.056 (−0.154, 0.043)	0.270	1.000	0.544	Regular
Potassium	mmol/L	592	−0.041 (−0.089, 0.006)	0.0848	1.000	0.343	Regular
Sodium	mmol./L	592	−0.139 (−0.485, 0.206)	0.428	1.000	0.656	Regular
Glucose	mg/dL	592	−0.833 (−2.158, 0.493)	0.218	1.000	0.535	Regular
Basophils (absolute count)	count	563	0.001 (−0.005, 0.006)	0.766	1.000	0.827	Regular
Eosinophils (absolute count)	count	563	−0.002 (−0.021, 0.017)	0.840	1.000	0.872	Regular
Haematocrit	pp	563	0.119 (−0.401, 0.640)	0.653	1.000	0.760	Regular
Haemoglobin	g/dL	563	−0.017 (−0.205, 0.171)	0.860	1.000	0.876	Boundary
Lymphocytes (absolute count)	count	563	−0.041 (−0.148, 0.066)	0.456	1.000	0.656	Regular
Mean corpuscular haemoglobin (MCH)	pg	563	0.087 (−0.304, 0.478)	0.661	1.000	0.760	Regular
Mean corpuscular haemoglobin concentration (MCHC)	g/dL	563	−0.101 (−0.288, 0.086)	0.290	1.000	0.544	Regular
Mean corpuscular volume (MCV)	fL	563	0.534 (−0.392, 1.460)	0.258	1.000	0.544	Regular
Mean platelet volume (MPV)	fL	562	−0.091 (−0.338, 0.155)	0.468	1.000	0.656	Regular
Monocytes (absolute count)	count	563	0.000 (−0.025, 0.025)	0.992	1.000	0.992	Boundary
Neutrophils (absolute count)	count	563	−0.111 (−0.322, 0.100)	0.302	1.000	0.544	Boundary
Platelets	/uL	563	−2609.389 (−11519.976, 6301.197)	0.565	1.000	0.710	Regular
Red blood cells	/uL	563	−28665.145 (−101390.829, 44060.539)	0.439	1.000	0.656	Regular
Red-cell distribution width (RDW)	pp	563	0.050 (−0.102, 0.203)	0.518	1.000	0.683	Regular
White blood cells	/uL	563	−148.220 (−426.720, 130.281)	0.296	1.000	0.544	Regular
ALT	%	592	−9.33 (−14.22, −4.17)	0.00054	0.0805	0.0176	Regular
ALP	%	592	−5.22 (−9.46, −0.78)	0.0217	0.765	0.167	Regular

Outcome	Scale	n	Difference (95% CI)	Raw p	BY adjusted p	BH comparison	State
AST	%	592	−8.01 (−12.31, −3.50)	0.00065	0.0805	0.0176	Regular
Direct bilirubin	%	592	9.73 (1.85, 18.22)	0.0148	0.729	0.159	Boundary
GGT	%	592	−7.27 (−13.97, −0.04)	0.0488	1.000	0.240	Regular
Total bilirubin	%	592	5.25 (−2.44, 13.56)	0.186	1.000	0.502	Regular
Ferritin	%	592	−5.71 (−17.45, 7.69)	0.385	1.000	0.630	Boundary
Serum iron	mcg/dL	592	0.997 (−4.568, 6.562)	0.725	1.000	0.799	Regular
Transferrin	mg/dL	592	−3.868 (−10.832, 3.096)	0.276	1.000	0.544	Regular
Homocysteine	%	592	−1.34 (−4.22, 1.62)	0.369	1.000	0.623	Regular
Cortisol	%	592	−1.98 (−7.21, 3.56)	0.476	1.000	0.656	Regular
Estradiol	%	592	2.29 (−3.20, 8.09)	0.420	1.000	0.656	Regular
Follicle-stimulating hormone (FSH)	%	592	3.73 (−8.05, 17.03)	0.551	1.000	0.708	Boundary
Free testosterone	%	592	−1.98 (−10.96, 7.90)	0.682	1.000	0.768	Regular
Luteinising hormone (LH)	%	592	15.02 (1.06, 30.91)	0.034	1.000	0.230	Boundary
Progesterone	%	592	6.28 (−4.01, 17.66)	0.241	1.000	0.544	Regular
Prolactin	%	592	−1.12 (−9.46, 7.97)	0.801	1.000	0.848	Boundary
Total testosterone (harmonised)	%	592	3.72 (−3.00, 10.91)	0.284	1.000	0.544	Regular
Lactate dehydrogenase (LDH)	%	592	−2.81 (−5.09, −0.48)	0.0184	0.759	0.166	Regular
Alpha-1 globulin	g/dL	592	−0.005 (−0.010, 0.001)	0.126	1.000	0.401	Regular
Alpha-2 globulin	g/dL	592	−0.012 (−0.027, 0.002)	0.0952	1.000	0.343	Boundary
Beta-1 globulin	g/dL	592	−0.012 (−0.021, −0.003)	0.0123	0.729	0.159	Regular
Beta-2 globulin	g/dL	592	−0.007 (−0.017, 0.004)	0.214	1.000	0.535	Regular
Gamma globulin	g/dL	592	−0.027 (−0.059, 0.005)	0.104	1.000	0.350	Boundary
Total protein	g/dL	592	−0.084 (−0.151, −0.018)	0.0127	0.729	0.159	Regular
Albumin	g/dL	592	−0.0434 (−0.0863, −0.0004)	0.0477	1.000	0.240	Regular
Pseudocholinesterase	%	592	−2.27 (−4.71, 0.23)	0.0743	1.000	0.334	Regular

Outcome	Scale	n	Difference (95% CI)	Raw p	BY adjusted p	BH comparison	State
Urea	%	592	−2.69 (−6.26, 1.02)	0.153	1.000	0.458	Regular
Creatinine	%	592	−1.48 (−3.56, 0.65)	0.172	1.000	0.489	Regular
Uric acid	%	592	−3.34 (−6.45, −0.12)	0.0425	1.000	0.240	Regular
FT4	ng/dL	592	−0.0047 (−0.0233, 0.0139)	0.621	1.000	0.760	Regular
FT3	pg/mL	592	0.0291 (−0.0337, 0.0919)	0.363	1.000	0.623	Regular
TSH	%	592	−7.23 (−14.96, 1.21)	0.0911	1.000	0.343	Boundary

Same primary ratio model and covariates as Table S5. BY ≤ 0.05 is the global criterion; BH is a comparison. No outcome met BY. Percent differences arise from log outcomes; identity outcomes use source-unit differences. All intervals are pointwise. Boundary denotes a converged model with an estimable contrast and boundary random-intercept variance; no estimator switch occurred. All 54 planned slots were retained. pp, percentage-point difference for identity-scale percentage outcomes. Differential-count effects use the recorded count scale listed in Table S3.

Table S7. Sensitivity estimates across the 13 specified variants.

Variant	Outcome	Scale	n	Difference (95% CI)	Reference n	State
Design-context adjustment	TSH	%	592	−8.18 (−15.79, 0.13)	592	Boundary
Design-context adjustment	FT4	ng/dL	592	−0.0017 (−0.0203, 0.0168)	592	Regular
Design-context adjustment	FT3	pg/mL	592	0.0263 (−0.0359, 0.0886)	592	Regular
Design-context adjustment	ALT	%	592	−9.37 (−14.22, −4.24)	592	Regular
Design-context adjustment	AST	%	592	−7.42 (−11.75, −2.88)	592	Regular
Design-context adjustment	GGT	%	592	−8.37 (−15.08, −1.13)	592	Regular
Design-context adjustment	ALP	%	592	−5.53 (−9.77, −1.10)	592	Regular
Design-context adjustment	Total bilirubin	%	592	6.50 (−1.29, 14.90)	592	Regular
Design-context adjustment	Direct bilirubin	%	592	11.14 (3.14, 19.76)	592	Boundary
Design-context adjustment	Albumin	g/dL	592	−0.0372 (−0.0800, 0.0057)	592	Regular
Design-context adjustment	Pseudocholinesterase	%	592	−2.76 (−5.23, −0.22)	592	Regular
Bioactivity adjusted for estimated total lipids	TSH	%	592	−3.60 (−11.92, 5.50)	592	Boundary
Bioactivity adjusted for estimated total lipids	FT4	ng/dL	592	−0.0040 (−0.0233, 0.0153)	592	Regular
Bioactivity adjusted for estimated total lipids	FT3	pg/mL	592	0.0294 (−0.0360, 0.0947)	592	Regular
Bioactivity adjusted for estimated total lipids	ALT	%	592	−8.38 (−13.51, −2.96)	592	Regular
Bioactivity adjusted for estimated total lipids	AST	%	592	−6.03 (−10.56, −1.28)	592	Regular
Bioactivity adjusted for estimated total lipids	GGT	%	592	−3.11 (−10.24, 4.60)	592	Regular
Bioactivity adjusted for estimated total lipids	ALP	%	592	−3.45 (−7.92, 1.23)	592	Regular

Variant	Outcome	Scale	n	Difference (95% CI)	Reference n	State
Bioactivity adjusted for estimated total lipids	Total bilirubin	%	592	2.00 (−5.72, 10.36)	592	Regular
Bioactivity adjusted for estimated total lipids	Direct bilirubin	%	592	1.32 (−5.98, 9.17)	592	Boundary
Bioactivity adjusted for estimated total lipids	Albumin	g/dL	592	−0.0145 (−0.0580, 0.0289)	592	Regular
Bioactivity adjusted for estimated total lipids	Pseudocholinesterase	%	592	−0.67 (−3.21, 1.93)	592	Regular
Bioactivity without lipid adjustment	TSH	%	592	0.03 (−8.46, 9.32)	592	Boundary
Bioactivity without lipid adjustment	FT4	ng/dL	592	−0.0023 (−0.0210, 0.0164)	592	Regular
Bioactivity without lipid adjustment	FT3	pg/mL	592	0.0262 (−0.0371, 0.0896)	592	Regular
Bioactivity without lipid adjustment	ALT	%	592	−6.35 (−11.47, −0.94)	592	Regular
Bioactivity without lipid adjustment	AST	%	592	−3.22 (−7.83, 1.62)	592	Regular
Bioactivity without lipid adjustment	GGT	%	592	2.31 (−5.19, 10.40)	592	Regular
Bioactivity without lipid adjustment	ALP	%	592	−1.51 (−5.97, 3.16)	592	Regular
Bioactivity without lipid adjustment	Total bilirubin	%	592	−0.96 (−8.31, 6.98)	592	Regular
Bioactivity without lipid adjustment	Direct bilirubin	%	592	−4.97 (−11.94, 2.54)	592	Boundary
Bioactivity without lipid adjustment	Albumin	g/dL	592	0.0190 (−0.0245, 0.0626)	592	Regular
Bioactivity without lipid adjustment	Pseudocholinesterase	%	592	1.04 (−1.53, 3.67)	592	Regular
Index using estimated total lipids	TSH	%	592	−7.12 (−14.93, 1.41)	592	Boundary
Index using estimated total lipids	FT4	ng/dL	592	−0.0051 (−0.0238, 0.0136)	592	Regular
Index using estimated total lipids	FT3	pg/mL	592	0.0291 (−0.0342, 0.0924)	592	Regular
Index using estimated total lipids	ALT	%	592	−9.35 (−14.27, −4.15)	592	Regular
Index using estimated total lipids	AST	%	592	−7.82 (−12.16, −3.26)	592	Regular
Index using estimated total lipids	GGT	%	592	−7.18 (−13.94, 0.12)	592	Regular

Variant	Outcome	Scale	n	Difference (95% CI)	Reference n	State
Index using estimated total lipids	ALP	%	592	−4.81 (−9.11, −0.31)	592	Regular
Index using estimated total lipids	Total bilirubin	%	592	4.60 (−3.11, 12.93)	592	Regular
Index using estimated total lipids	Direct bilirubin	%	592	8.75 (0.86, 17.25)	592	Boundary
Index using estimated total lipids	Albumin	g/dL	592	−0.0450 (−0.0883, −0.0017)	592	Regular
Index using estimated total lipids	Pseudocholinesterase	%	592	−2.21 (−4.67, 0.32)	592	Regular
Bioactivity adjusted for lipid-component sum	TSH	%	592	−3.42 (−11.74, 5.68)	592	Boundary
Bioactivity adjusted for lipid-component sum	FT4	ng/dL	592	−0.0037 (−0.0230, 0.0156)	592	Regular
Bioactivity adjusted for lipid-component sum	FT3	pg/mL	592	0.0294 (−0.0358, 0.0946)	592	Regular
Bioactivity adjusted for lipid-component sum	ALT	%	592	−8.32 (−13.44, −2.91)	592	Regular
Bioactivity adjusted for lipid-component sum	AST	%	592	−6.03 (−10.54, −1.30)	592	Regular
Bioactivity adjusted for lipid-component sum	GGT	%	592	−2.96 (−10.10, 4.74)	592	Regular
Bioactivity adjusted for lipid-component sum	ALP	%	592	−3.61 (−8.05, 1.06)	592	Regular
Bioactivity adjusted for lipid-component sum	Total bilirubin	%	592	2.20 (−5.51, 10.54)	592	Regular
Bioactivity adjusted for lipid-component sum	Direct bilirubin	%	592	1.28 (−5.96, 9.09)	592	Boundary
Bioactivity adjusted for lipid-component sum	Albumin	g/dL	592	−0.0120 (−0.0555, 0.0315)	592	Regular
Bioactivity adjusted for lipid-component sum	Pseudocholinesterase	%	592	−0.60 (−3.13, 2.00)	592	Boundary
Alternative geographic adjustment	TSH	%	592	−8.70 (−16.14, −0.61)	592	Regular

Variant	Outcome	Scale	n	Difference (95% CI)	Reference n	State
Alternative geographic adjustment	FT4	ng/dL	592	−0.0021 (−0.0299, 0.0258)	592	Regular
Alternative geographic adjustment	FT3	pg/mL	592	0.0283 (−0.0555, 0.1121)	592	Regular
Alternative geographic adjustment	ALT	%	592	−8.41 (−13.10, −3.47)	592	Regular
Alternative geographic adjustment	AST	%	592	−7.05 (−11.30, −2.60)	592	Regular
Alternative geographic adjustment	GGT	%	592	−5.68 (−12.35, 1.49)	592	Regular
Alternative geographic adjustment	ALP	%	592	−4.38 (−8.24, −0.36)	592	Regular
Alternative geographic adjustment	Total bilirubin	%	592	3.47 (−7.06, 15.18)	592	Regular
Alternative geographic adjustment	Direct bilirubin	%	592	9.24 (−0.77, 20.26)	592	Regular
Alternative geographic adjustment	Albumin	g/dL	592	−0.0508 (−0.0947, −0.0069)	592	Regular
Alternative geographic adjustment	Pseudocholinesterase	%	592	−1.56 (−4.03, 0.98)	592	Regular
BMI omitted	TSH	%	592	−7.78 (−15.45, 0.60)	592	Boundary
BMI omitted	FT4	ng/dL	592	−0.0034 (−0.0220, 0.0152)	592	Regular
Current smokers excluded	TSH	%	590	−7.12 (−14.87, 1.34)	592	Boundary
Current smokers excluded	FT4	ng/dL	590	−0.0048 (−0.0235, 0.0138)	592	Regular
Upper exposure tail excluded	TSH	%	586	−8.06 (−16.01, 0.64)	592	Boundary
Upper exposure tail excluded	FT4	ng/dL	586	−0.0009 (−0.0201, 0.0182)	592	Regular
Alcohol omitted with matched sample	TSH	%	592	−7.78 (−15.45, 0.58)	592	Boundary
Alcohol omitted with matched sample	FT4	ng/dL	592	−0.0034 (−0.0219, 0.0151)	592	Regular
Alcohol omitted with matched sample	FT3	pg/mL	592	0.0275 (−0.0350, 0.0900)	592	Regular
Alcohol omitted with matched sample	ALT	%	592	−9.24 (−14.10, −4.10)	592	Regular
Alcohol omitted with matched sample	AST	%	592	−7.32 (−11.67, −2.77)	592	Regular

Variant	Outcome	Scale	n	Difference (95% CI)	Reference n	State
Alcohol omitted with matched sample	GGT	%	592	−7.13 (−13.82, 0.07)	592	Regular
Alcohol omitted with matched sample	ALP	%	592	−5.00 (−9.23, −0.57)	592	Regular
Alcohol omitted with matched sample	Total bilirubin	%	592	5.73 (−1.97, 14.04)	592	Regular
Alcohol omitted with matched sample	Direct bilirubin	%	592	9.88 (2.02, 18.34)	592	Boundary
Alcohol omitted with matched sample	Albumin	g/dL	592	−0.0414 (−0.0842, 0.0014)	592	Regular
Alcohol omitted with matched sample	Pseudocholinesterase	%	592	−2.29 (−4.72, 0.19)	592	Regular
Alcohol omitted with expanded sample	TSH	%	594	−7.45 (−15.12, 0.92)	592	Boundary
Alcohol omitted with expanded sample	FT4	ng/dL	594	−0.0036 (−0.0221, 0.0148)	592	Regular
Alcohol omitted with expanded sample	FT3	pg/mL	594	0.0258 (−0.0365, 0.0880)	592	Regular
Alcohol omitted with expanded sample	ALT	%	594	−9.17 (−14.02, −4.05)	592	Regular
Alcohol omitted with expanded sample	AST	%	594	−7.01 (−11.36, −2.45)	592	Regular
Alcohol omitted with expanded sample	GGT	%	594	−7.71 (−14.34, −0.57)	592	Regular
Alcohol omitted with expanded sample	ALP	%	594	−4.86 (−9.09, −0.43)	592	Regular
Alcohol omitted with expanded sample	Total bilirubin	%	594	5.63 (−2.03, 13.89)	592	Regular
Alcohol omitted with expanded sample	Direct bilirubin	%	594	9.63 (1.82, 18.04)	592	Boundary

Variant	Outcome	Scale	n	Difference (95% CI)	Reference n	State
Alcohol omitted with expanded sample	Albumin	g/dL	594	−0.0405 (−0.0830, 0.0021)	592	Regular
Alcohol omitted with expanded sample	Pseudocholinesterase	%	594	−2.22 (−4.64, 0.26)	592	Regular
Deprivation score added	TSH	%	592	−7.36 (−15.07, 1.04)	592	Boundary
Deprivation score added	FT4	ng/dL	592	−0.0048 (−0.0234, 0.0138)	592	Regular
Deprivation score added	FT3	pg/mL	592	0.0288 (−0.0340, 0.0916)	592	Regular
Deprivation score added	ALT	%	592	−9.37 (−14.25, −4.22)	592	Regular
Deprivation score added	AST	%	592	−8.06 (−12.35, −3.56)	592	Regular
Deprivation score added	GGT	%	592	−7.32 (−14.02, −0.10)	592	Regular
Deprivation score added	ALP	%	592	−5.23 (−9.48, −0.79)	592	Regular
Deprivation score added	Total bilirubin	%	592	5.23 (−2.46, 13.54)	592	Regular
Deprivation score added	Direct bilirubin	%	592	9.66 (1.78, 18.15)	592	Boundary
Deprivation score added	Albumin	g/dL	592	−0.0437 (−0.0865, −0.0008)	592	Regular
Deprivation score added	Pseudocholinesterase	%	592	−2.28 (−4.72, 0.23)	592	Regular
Medication-free restriction	TSH	%	464	−4.06 (−13.47, 6.38)	592	Boundary
Medication-free restriction	FT4	ng/dL	464	0.0022 (−0.0201, 0.0245)	592	Regular
Medication-free restriction	FT3	pg/mL	464	0.0558 (−0.0216, 0.1333)	592	Regular
Medication-free restriction	ALT	%	464	−11.98 (−17.67, −5.90)	592	Regular
Medication-free restriction	AST	%	464	−10.34 (−15.21, −5.19)	592	Regular
Medication-free restriction	GGT	%	464	−7.88 (−15.74, 0.70)	592	Regular
Medication-free restriction	ALP	%	464	−5.99 (−11.07, −0.62)	592	Regular
Medication-free restriction	Total bilirubin	%	464	8.46 (−1.14, 19.00)	592	Regular
Medication-free restriction	Direct bilirubin	%	464	13.92 (4.24, 24.50)	592	Boundary
Medication-free restriction	Albumin	g/dL	464	−0.0503 (−0.1011, 0.0005)	592	Regular

Variant	Outcome	Scale	n	Difference (95% CI)	Reference n	State
Medication-free restriction	Pseudocholinesterase	%	464	−2.92 (−5.83, 0.07)	592	Boundary

S8 defines each variant and its endpoint scope. Ten variants cover all 11 primary and secondary endpoints; BMI omission, smoker exclusion and upper-tail exclusion cover only TSH and FT4. These estimation-only comparisons have no p-value family. Reference n identifies the full principal reference population; matched restricted principal refits are provided in Table S8. Boundary marks retained singular mixed models. Effects are per doubling of the exposure used in that variant; separate-term lipid models condition on their lipid term.

Table S8. Matched principal-model refits for restricted sensitivity analyses.

Reference label	Outcome	Scale	n	Difference (95% CI)	State
Matched sample with current smokers excluded	TSH	%	590	−7.12 (−14.87, 1.34)	Boundary
Matched sample with current smokers excluded	FT4	ng/dL	590	−0.0048 (−0.0235, 0.0138)	Regular
Matched sample with upper exposure tail excluded	TSH	%	586	−8.06 (−16.01, 0.64)	Boundary
Matched sample with upper exposure tail excluded	FT4	ng/dL	586	−0.0009 (−0.0201, 0.0182)	Regular
Matched medication-free sample	TSH	%	464	−4.06 (−13.47, 6.38)	Boundary
Matched medication-free sample	FT4	ng/dL	464	0.0022 (−0.0201, 0.0245)	Regular
Matched medication-free sample	FT3	pg/mL	464	0.0558 (−0.0216, 0.1333)	Regular
Matched medication-free sample	ALT	%	464	−11.98 (−17.67, −5.90)	Regular
Matched medication-free sample	AST	%	464	−10.34 (−15.21, −5.19)	Regular
Matched medication-free sample	GGT	%	464	−7.88 (−15.74, 0.70)	Regular
Matched medication-free sample	ALP	%	464	−5.99 (−11.07, −0.62)	Regular
Matched medication-free sample	Total bilirubin	%	464	8.46 (−1.14, 19.00)	Regular
Matched medication-free sample	Direct bilirubin	%	464	13.92 (4.24, 24.50)	Boundary
Matched medication-free sample	Albumin	g/dL	464	−0.0503 (−0.1011, 0.0005)	Regular
Matched medication-free sample	Pseudocholinesterase	%	464	−2.92 (−5.83, 0.07)	Boundary

These 15 refits separate changes in sample composition from changes in specification. They are estimates on the sensitivity-specific participants, not additional hypothesis tests. The full principal results remain in Table S5. Effects are per doubling of the primary index.

Table S9. Alternative lipid and geographic models for all 54 outcomes.

Outcome	Scale	n in each	Bioactivity adjusted for estimated total lipids	Bioactivity adjusted for lipid-component sum	Alternative geographic adjustment
Calcium	mg/dL	592	0.016 (−0.044, 0.076)	0.017 (−0.043, 0.077)	−0.016 (−0.073, 0.041)
Chloride	mmol/L	592	−0.343 (−0.742, 0.057)	−0.342 (−0.741, 0.057)	−0.184 (−0.619, 0.251)
Phosphorus	mg/dL	592	−0.049 (−0.152, 0.054)	−0.049 (−0.152, 0.053)	−0.052 (−0.164, 0.061)
Potassium	mmol/L	592	−0.047 (−0.096, 0.002)	−0.048 (−0.097, 0.000)	−0.039 (−0.094, 0.016)
Sodium	mmol./L	592	−0.177 (−0.537, 0.182)	−0.158 (−0.517, 0.201)	−0.106 (−0.445, 0.233)
Glucose	mg/dL	592	−0.756 (−2.134, 0.623)	−0.723 (−2.100, 0.653)	−0.760 (−2.379, 0.859)
Basophils	count	563	0.002 (−0.004, 0.007)	0.002 (−0.004, 0.007)	0.000 (−0.005, 0.004)
Eosinophils	count	563	−0.002 (−0.022, 0.018)	−0.002 (−0.022, 0.018)	−0.005 (−0.023, 0.013)
Haematocrit	pp	563	0.324 (−0.210, 0.859)	0.325 (−0.208, 0.858)	0.055 (−0.630, 0.740)
Haemoglobin	g/dL	563	0.061 (−0.132, 0.255) *	0.062 (−0.131, 0.256) *	−0.033 (−0.305, 0.239)
Lymphocytes	count	563	0.011 (−0.099, 0.121)	0.010 (−0.099, 0.120)	−0.046 (−0.170, 0.079)
MCH	pg	563	0.124 (−0.282, 0.529)	0.127 (−0.278, 0.532)	0.063 (−0.533, 0.659)
MCHC	g/dL	563	−0.100 (−0.294, 0.094)	−0.098 (−0.291, 0.096)	−0.093 (−0.461, 0.275)
MCV	fL	563	0.605 (−0.354, 1.564)	0.613 (−0.345, 1.570)	0.451 (−0.647, 1.548)
MPV	fL	562	−0.167 (−0.422, 0.087)	−0.172 (−0.426, 0.082)	−0.111 (−0.352, 0.129)
Monocytes	count	563	0.001 (−0.025, 0.027) *	0.001 (−0.026, 0.027) *	0.003 (−0.032, 0.037)
Neutrophils	count	563	−0.055 (−0.274, 0.164) *	−0.064 (−0.282, 0.155) *	−0.106 (−0.312, 0.099)
Platelets	/uL	563	1166.893 (−8020.950, 10354.735)	1106.883 (−8060.172, 10273.938)	−2554.064 (−11805.232, 6697.104)
Red blood cells	/uL	563	−8383.613 (−83700.391, 66933.165)	−8716.168 (−83883.188, 66450.851)	−28513.868 (−131270.742, 74243.005)
RDW	pp	563	0.044 (−0.114, 0.202)	0.046 (−0.112, 0.203)	0.037 (−0.232, 0.307)
White blood cells	/uL	563	−36.367 (−324.324, 251.589)	−46.028 (−333.039, 240.982)	−154.803 (−434.718, 125.112)
ALT	%	592	−8.38 (−13.51, −2.96)	−8.32 (−13.44, −2.91)	−8.41 (−13.10, −3.47)
ALP	%	592	−3.45 (−7.92, 1.23)	−3.61 (−8.05, 1.06)	−4.38 (−8.24, −0.36)

Outcome	Scale	n in each	Bioactivity adjusted for estimated total lipids	Bioactivity adjusted for lipid-component sum	Alternative geographic adjustment
AST	%	592	−6.03 (−10.56, −1.28)	−6.03 (−10.54, −1.30)	−7.05 (−11.30, −2.60)
Direct bilirubin	%	592	1.32 (−5.98, 9.17) *	1.28 (−5.96, 9.09) *	9.24 (−0.77, 20.26)
GGT	%	592	−3.11 (−10.24, 4.60)	−2.96 (−10.10, 4.74)	−5.68 (−12.35, 1.49)
Total bilirubin	%	592	2.00 (−5.72, 10.36)	2.20 (−5.51, 10.54)	3.47 (−7.06, 15.18)
Ferritin	%	592	−1.31 (−14.07, 13.35) *	−1.39 (−14.11, 13.21) *	−2.64 (−15.14, 11.69)
Serum iron	mcg/dL	592	1.432 (−4.372, 7.237)	1.655 (−4.140, 7.450)	1.768 (−5.352, 8.888)
Transferrin	mg/dL	592	0.349 (−6.755, 7.453)	0.491 (−6.604, 7.585)	−3.269 (−11.309, 4.771)
Homocysteine	%	592	−1.57 (−4.54, 1.50)	−1.52 (−4.49, 1.54)	−1.38 (−4.79, 2.16)
Cortisol	%	592	−1.47 (−6.94, 4.31)	−1.59 (−7.04, 4.18)	−2.55 (−8.03, 3.27)
Estradiol	%	592	3.29 (−2.46, 9.38)	3.27 (−2.47, 9.34)	2.92 (−3.44, 9.70)
Follicle-stimulating hormone (FSH)	%	592	5.19 (−7.26, 19.32) *	5.57 (−6.91, 19.71) *	4.64 (−7.47, 18.34)
Free testosterone	%	592	−4.04 (−13.13, 6.01)	−4.09 (−13.17, 5.93)	−3.03 (−12.73, 7.75)
Luteinising hormone (LH)	%	592	13.24 (−1.10, 29.65) *	13.79 (−0.57, 30.23) *	14.33 (−3.59, 35.58)
Progesterone	%	592	6.17 (−4.49, 18.01)	6.00 (−4.62, 17.81)	5.39 (−5.42, 17.44)
Prolactin	%	592	−0.16 (−8.94, 9.46) *	−0.06 (−8.83, 9.55) *	−0.37 (−8.81, 8.84)
Total testosterone (harmonised)	%	592	5.57 (−1.52, 13.18)	5.70 (−1.39, 13.30)	4.42 (−3.79, 13.33)
Lactate dehydrogenase (LDH)	%	592	−1.99 (−4.37, 0.45)	−2.00 (−4.38, 0.43)	−3.17 (−5.65, −0.63)
Alpha-1 globulin	g/dL	592	−0.002 (−0.009, 0.004)	−0.003 (−0.009, 0.004)	−0.006 (−0.011, 0.000)
Alpha-2 globulin	g/dL	592	−0.005 (−0.020, 0.010) *	−0.005 (−0.020, 0.010) *	−0.010 (−0.027, 0.007)
Beta-1 globulin	g/dL	592	−0.006 (−0.015, 0.003)	−0.006 (−0.015, 0.003)	−0.013 (−0.023, −0.004)
Beta-2 globulin	g/dL	592	−0.006 (−0.017, 0.005)	−0.006 (−0.017, 0.005)	−0.008 (−0.021, 0.005)
Gamma globulin	g/dL	592	−0.031 (−0.065, 0.003) *	−0.032 (−0.065, 0.002) *	−0.026 (−0.061, 0.009)
Total protein	g/dL	592	−0.039 (−0.107, 0.028)	−0.036 (−0.104, 0.031)	−0.094 (−0.174, −0.014)

Outcome	Scale	n in each	Bioactivity adjusted for estimated total lipids	Bioactivity adjusted for lipid-component sum	Alternative geographic adjustment
Albumin	g/dL	592	−0.0145 (−0.0580, 0.0289)	−0.0120 (−0.0555, 0.0315)	−0.0508 (−0.0947, −0.0069)
Pseudocholinesterase	%	592	−0.67 (−3.21, 1.93)	−0.60 (−3.13, 2.00) *	−1.56 (−4.03, 0.98)
Urea	%	592	−2.54 (−6.27, 1.33)	−2.46 (−6.18, 1.41)	−2.43 (−6.01, 1.28)
Creatinine	%	592	−0.76 (−2.92, 1.46)	−0.69 (−2.86, 1.53)	−1.63 (−3.88, 0.68)
Uric acid	%	592	−2.54 (−5.80, 0.83)	−2.47 (−5.73, 0.90)	−3.45 (−6.88, 0.11)
FT4	ng/dL	592	−0.0040 (−0.0233, 0.0153)	−0.0037 (−0.0230, 0.0156)	−0.0021 (−0.0299, 0.0258)
FT3	pg/mL	592	0.0294 (−0.0360, 0.0947)	0.0294 (−0.0358, 0.0946)	0.0283 (−0.0555, 0.1121)
TSH	%	592	−3.60 (−11.92, 5.50) *	−3.42 (−11.74, 5.68) *	−8.70 (−16.14, −0.61)

Cells show differences with pointwise 95% confidence intervals. The two lipid models include $\log_2$ non-normalised bioactivity and the indicated $\log_2$ lipid measure as freely estimated terms. Alternative geographic adjustment uses the primary index with sampling-cluster fixed effects and municipality CR2/Satterthwaite inference. Each comparison uses the same participants as its principal model. The 33 estimates for the 11 primary and secondary outcomes also appear in Table S7. An asterisk marks retained boundary random-intercept variance. No additional p-value family applies. Lipid-adjusted contrasts concern bioactivity doubling at fixed modelled lipids; geographic contrasts concern primary-index doubling. Units and abbreviations follow Table S3.

Table S10. Exploratory thyroid spline and interaction tests.

Outcome	Test family	F	Numerator df	Denominator df	Raw p	BH adjusted p
TSH	spline global	1.754	2	492.02	0.174	0.348
TSH	spline nonlinear	0.603	1	504.10	0.438	0.438
TSH	sex interaction	0.821	1	575.60	0.365	0.731
FT4	spline global	0.748	2	577.33	0.474	0.474
FT4	spline nonlinear	1.269	1	579.98	0.261	0.438
FT4	sex interaction	0.062	1	576.22	0.803	0.803

Each of global spline, nonlinear component and exposure-by-sex interaction forms a separate BH family of two. All models use n = 592. None met its adjusted threshold. These tests do not replace the two Holm-controlled linear thyroid tests.

Table S11. Sex-specific thyroid slopes from pooled interaction models.

Outcome	Sex contrast	Scale	Pooled model n	Difference (95% CI)	State
TSH	Women	%	592	−10.55 (−20.48, 0.63)	Boundary
TSH	Men	%	592	−3.28 (−14.66, 9.61)	Boundary
FT4	Women	ng/dL	592	−0.0026 (−0.0276, 0.0225)	Regular
FT4	Men	ng/dL	592	−0.0071 (−0.0336, 0.0195)	Regular

Both sex-specific slopes are linear contrasts of the same pooled interaction model. The n column is the pooled model size, not a sex-stratum count. Intervals are pointwise; differing subgroup significance labels are not a test of interaction. Each slope concerns a doubling of the primary index.

Table S12. Aggregate participant- and municipality-deletion diagnostics.

Outcome	Deletion unit	Available refits	Effect range	Maximum $|\Delta\beta|$	Maximum $|\Delta\beta|$ / full SE
TSH	participant	592/592	−7.9788 to −6.3902	0.00896	0.202
TSH	municipality	63/63	−8.8003 to −5.9868	0.01712	0.386
FT4	participant	592/592	−0.0068 to −0.0021	0.00262	0.277
FT4	municipality	63/63	−0.0089 to 0.0043	0.00897	0.948

TSH effect ranges are percent differences; FT4 ranges are ng/dL differences. $\Delta\beta$ is measured on the fitted outcome scale, and full SE is the original Kenward–Roger standard error. All 1,310 refits are diagnostic, with no resulting exclusions or new hypothesis tests. No individual or municipality identifiers are disclosed. These diagnostics concern TSH and FT4 only.

Table S13. Residual and support summary for every exploratory principal model.

Outcome	n	Residual SD	Maximum |standardised residual|	Count |residual| > 3	State
Albumin	592	0.2532	3.947	2	Regular
Alpha-1 globulin	592	0.0347	4.339	5	Regular
Alpha-2 globulin	592	0.0865	5.883	2	Boundary
Urea	592	0.2216	3.593	5	Regular
Basophils (absolute count)	563	0.0311	6.882	9	Regular
Beta-1 globulin	592	0.0541	4.928	4	Regular
Beta-2 globulin	592	0.0620	7.072	8	Regular
Total bilirubin	592	0.4511	3.141	3	Regular
Direct bilirubin	592	0.4453	3.039	1	Boundary
Calcium	592	0.3437	3.180	2	Regular
Chloride	592	2.2858	2.635	0	Regular
Cortisol	592	0.3218	3.814	3	Regular
Creatinine	592	0.1261	7.701	6	Regular
Haematocrit	563	2.9767	4.197	5	Regular
Haemoglobin	563	1.0843	4.432	7	Boundary
Eosinophils (absolute count)	563	0.1115	5.766	9	Regular
Estradiol	592	0.3234	6.510	10	Regular
Ferritin	592	0.7942	3.711	3	Boundary
ALP	592	0.2691	3.550	1	Regular
Phosphorus	592	0.5863	4.561	3	Regular
Follicle-stimulating hormone (FSH)	592	0.7208	6.308	11	Boundary
FT3	592	0.3695	4.591	14	Regular
FT4	592	0.1090	3.506	5	Regular
Gamma globulin	592	0.1930	3.895	2	Boundary

Outcome	n	Residual SD	Maximum |standardised residual|	Count |residual| > 3	State
GGT	592	0.4439	5.853	7	Regular
Glucose	592	7.8015	5.527	4	Regular
White blood cells	563	1602.8037	4.225	7	Regular
Red blood cells	563	419298.8636	4.348	9	Regular
AST	592	0.2833	7.574	7	Regular
ALT	592	0.3265	4.207	11	Regular
Lactate dehydrogenase (LDH)	592	0.1400	4.206	4	Regular
Luteinising hormone (LH)	592	0.7730	5.936	9	Boundary
Lymphocytes (absolute count)	563	0.6136	3.955	7	Regular
Mean corpuscular haemoglobin (MCH)	563	2.2477	4.657	17	Regular
Mean corpuscular haemoglobin concentration (MCHC)	563	1.0700	4.319	9	Regular
Mean corpuscular volume (MCV)	563	5.2929	4.822	15	Regular
Monocytes (absolute count)	563	0.1452	5.521	4	Boundary
Mean platelet volume (MPV)	562	1.4155	3.918	6	Regular
Neutrophils (absolute count)	563	1.2170	4.660	9	Boundary
Homocysteine	592	0.1729	3.559	2	Regular
Platelets	563	51218.1960	4.386	4	Regular
Potassium	592	0.2758	3.323	3	Regular
Progesterone	592	0.5977	7.162	5	Regular
Prolactin	592	0.5260	3.777	4	Boundary
Total protein	592	0.3951	3.017	1	Regular
Pseudocholinesterase	592	0.1505	5.667	5	Regular
Red-cell distribution width (RDW)	563	0.8727	6.206	10	Regular
Serum iron	592	33.1160	4.471	4	Regular

Outcome	n	Residual SD	Maximum |standardised residual|	Count |residual| > 3	State
Sodium	592	2.0384	2.895	0	Regular
Free testosterone	592	0.5626	7.105	9	Regular
Transferrin	592	40.7693	4.488	3	Regular
TSH	592	0.5200	5.391	6	Boundary
Uric acid	592	0.1936	5.150	5	Regular
Total testosterone (harmonised)	592	0.3961	3.227	2	Regular

Residual SD is on the modelled outcome scale. Standardised residuals are conditional model residuals divided by residual SD; these flags do not measure causal importance or independently establish influence. No automatic outlier exclusions were performed. All 54 models yielded estimable exposure contrasts; boundary models were retained.

Table S14. Clinical data handling.

Data item	Recorded information	Analytical treatment
Primary exposure	Source bioactivity divided by the lipid-component sum	Primary index and $\log_2$ per-doubling contrast as defined in S3
Alternative lipids	Complete Akins estimate and TC + TG + PL component sum	Prespecified ratio and separate-term comparisons; equations in S3
Albumin	One albumin field selected consistently	One source field retained throughout; no averaging or duplicate-column sensitivity
Bilirubin	Total equals direct plus indirect in all 600 records	Total and direct retained; indirect excluded as redundant
Protein fractions	Absolute concentrations recorded in the clinical dataset	Source values retained without reconstruction from total protein
Mean platelet volume	Invalid measurement	Treated as missing for this outcome only
Eosinophils	Two recorded zeros	Retained on the identity scale without a pseudocount
Total testosterone	60 values in ng/mL; 540 in nmol/L	ng/mL values multiplied by 3.47; existing nmol/L values unchanged
TSH	Study clinical documentation specifies mIU/L	Reported in mIU/L; numerical values unchanged
Differential counts	Original laboratory count notation retained in Table S3	Recorded numerical count scale used consistently for distributions and effects

Table S3 contains the complete outcome inventory; S3–S5 describe exposure calculation, unit harmonisation and outcome-specific missingness.

Table S15. Reference-interval and low-ALT summaries for aminotransferase activities.

Measure	DR-CALUX quantified (n = 594)	Principal analyses (n = 592)
ALT within 0–59 U/L	575 (96.8%)	573 (96.8%)
AST within 0–40 U/L	576 (97.0%)	574 (97.0%)
Both within these intervals	571 (96.1%)	569 (96.1%)
ALT <17 U/L (literature threshold)	22 (3.7%)	22 (3.7%)

ALT and AST were compared with the intervals printed in an available SPES laboratory report: ALT 0–59 U/L and AST 0–40 U/L, including both endpoints. The example report was for an adult man; these intervals were applied as common descriptive benchmarks rather than sex-specific diagnostic classifications. They did not determine original model eligibility; the reviewer-requested joint-benchmark restriction is reported separately in Table S17. The ALT <17 U/L row corresponds to the literature threshold discussed in the main article; it is not a laboratory lower reference limit.

Table S16. Principal DR-CALUX analytical sample versus nonselected SPES reference-cohort participants.

Characteristic	Principal DR sample (n=592)	Nonselected participants (n=3,589)	SMD
Age, years	34 [27–42]; n=592	35 [27–43]; n=3,589	−0.048
BMI, kg/m²	24.44 [22.43–27.34]; n=592	24.69 [22.22–27.76]; n=3,588	−0.130
Alcohol, g/day	0.15 [0.15–6.07]; n=592	1.25 [0.15–7.38]; n=3,539	−0.170
ALT, U/L	35 [30–40]; n=592	35 [30–40]; n=3,589	0.023
AST, U/L	23.00 [19.00–28.00]; n=592	22.00 [19.00–27.00]; n=3,589	0.065
TSH, mIU/L	1.78 [1.30–2.43]; n=592	1.78 [1.27–2.46]; n=3,589	−0.026
FT4, ng/dL	1.05 [0.98–1.14]; n=592	1.06 [0.99–1.15]; n=3,589	−0.062
Glucose, mg/dL	77 [72–84]; n=592	77 [72–83]; n=3,589	−0.074
Total cholesterol, mg/dL	185 [163–210]; n=592	188 [165–213]; n=3,589	−0.100
Triglycerides, mg/dL	85 [68–115]; n=592	92 [72–126]; n=3,589	−0.088
Phospholipids, mg/dL	254 [229–282]; n=592	256 [229–286]; n=3,587	−0.089
Women	298/592 (50.34%)	1,959/3,589 (54.58%)	−0.085
Current smoking	2/592 (0.34%)	1,274/3,539 (36.00%)	−1.043
Recent/current medication	126/590 (21.36%)	1,251/3,587 (34.88%)	−0.304
Low-impact area	120/592 (20.27%)	477/3,589 (13.29%)	0.188
Medium-impact area	237/592 (40.03%)	956/3,589 (26.64%)	0.287
High-impact area	235/592 (39.70%)	2,156/3,589 (60.07%)	−0.416

Continuous values are median [IQR]; categorical values are n/available N (%). Missing N equals group N minus available N. Continuous SMDs use the mean difference divided by the square root of the average within-group variances; categorical SMDs use the corresponding pooled Bernoulli variance. The sign is DR minus nonselected participants. The DR column contains the same 592 participants as the principal-model column in Table 1; medication percentages here use 590 available responses, whereas Table 1 uses 592 and displays two unknown responses separately. The reference group contains 3,589 participants outside the original 600-person CALUX subgroup. Seven other CALUX-selected members of the 4,188-person reference cohort are excluded from both columns (592 + 3,589 = 4,181). The table describes composition, not proof of representativeness or an estimate of selection bias.

Table S17. Joint aminotransferase benchmark-restricted sensitivity.

Outcome	Original sample, n=592<br>Difference, % (95% CI)	Joint benchmark-restricted subset, n=569<br>Difference, % (95% CI)
ALT	−9.33 (−14.22 to −4.17)	−6.54 (−11.20 to −1.64)
AST	−8.01 (−12.31 to −3.50)	−5.06 (−8.56 to −1.44)

Both outcomes were required to be positive and at or below the common upper benchmarks: ALT ≤59 U/L and AST ≤40 U/L. These were drawn from an example report for an adult man, not verified sex-specific diagnostic limits. Of 592 principal participants, 19 exceeded the ALT limit, 18 the AST limit and 14 both; the joint restriction excluded 23 and retained 569. The two models retained the original transformations, exposure, covariates, full-precision age/BMI knots and REML/Kenward–Roger method. Effects are percentage differences in conditional geometric means per doubling of the primary index; intervals are pointwise. Outcome-range restriction changes the analysed population and does not establish clinical normality or absence of liver disease.

Table S18. Pooled DR-CALUX-by-environmental-impact-area analyses for ALT and AST.

A. Joint two-degree-of-freedom interaction tests (n=592).

Outcome	F	Numerator df	Denominator df	Unadjusted p	BH-adjusted p
ALT	0.199	2	559.18	0.8196	0.8196
AST	1.106	2	536.04	0.3317	0.6635

B. Area-specific slopes from the pooled interaction models.

Impact area	n	Sampling clusters	Municipalities	ALT, % difference (95% CI)	AST, % difference (95% CI)
Low	120	2	19	−7.53 (−18.48 to 4.89)	−6.51 (−16.19 to 4.29)
Medium	237	5	24	−11.55 (−19.59 to −2.70)	−12.57 (−19.37 to −5.20)
High	235	4	20	−8.55 (−15.60 to −0.92)	−5.47 (−11.81 to 1.34)

C. Exposure and design support within each area.

Area	n	Clusters	Municipalities	$\log_2$ index: minimum	Q1	Median	Q3	Maximum
Low	120	2	19	3.634	4.978	5.310	5.653	6.637
Medium	237	5	24	4.190	5.213	5.526	5.885	6.653
High	235	4	20	1.961	5.018	5.326	5.665	6.919

One pooled model per outcome retained the main exposure and area effects, two exposure-by-area coefficients, original covariates and spline knots, and sampling-cluster random intercept. BH covers the two omnibus p values only. Area-specific slopes use linear contrasts and the full Kenward–Roger-adjusted covariance matrix, with contrast-specific degrees of freedom and pointwise intervals. Both pooled fits were available and nonboundary. Participant counts are not independent-cluster counts; the low-impact area has only two clusters. Non-significant interactions do not demonstrate identical associations, and differences in area-specific significance labels do not establish interaction. All slopes and support are reported irrespective of statistical support.

Table S19. Variance and inferential diagnostics for the four focal original models.

Outcome	Random-intercept variance	Residual variance	Conditional ICC	Exposure KR df	Boundary fit
TSH	≈0	0.270443	≈0	541.03	Yes
FT4	0.001204	0.011880	0.0920	580.88	No
ALT	0.003200	0.106614	0.0291	575.68	No
AST	0.001161	0.080260	0.0143	566.31	No

All models: n=592, 11 sampling clusters and 63 municipalities. Variances are on each model's outcome scale (natural-log for TSH/ALT/AST; $(ng/dL)^2$ for FT4 variance). Conditional ICC = random-intercept variance/(random-intercept + residual variance). KR denotes Kenward–Roger; numerically negligible TSH random variance is shown as approximately zero. Large contrast-specific df do not imply that hundreds of independent sampling clusters were sampled. Residual diagnostics for all 54 outcomes remain in Table S13. Maximum absolute standardised residuals for TSH/FT4/ALT/AST were 5.39/3.51/4.21/7.57, with 6/5/11/7 exceeding three. No such flag caused exclusion. The existing alternative geographic model uses sampling-cluster fixed effects and municipality CR2/Satterthwaite inference (exposure df=21.42); it changes both mean and covariance assumptions rather than independently validating the mixed model.

Table S20. ALT/AST participant-, municipality- and sampling-cluster-deletion diagnostics.

Outcome	Deleted unit	Available / attempted	Effect range, %	Maximum $\|\Delta\beta\|$ / full SE	Boundary refits
ALT	Participant	592/592	−10.03 to −8.84	0.273	0
ALT	Municipality	63/63	−10.60 to −8.61	0.499	0
ALT	Sampling cluster	11/11	−10.33 to −8.59	0.391	0
AST	Participant	592/592	−8.83 to −6.88	0.502	0
AST	Municipality	63/63	−9.36 to −7.32	0.607	1
AST	Sampling cluster	11/11	−8.95 to −6.58	0.634	1

All 1,332 planned refits were available; no deletion reversed an exposure coefficient. Ranges concern point estimates per doubling of the primary index. Full SE is the original-model Kenward–Roger standard error; $|\Delta\beta|$/full SE is descriptive, not exact deletion DFBETAS. These deletion fits did not recalculate a new KR covariance for each refit or produce new tests/intervals. One AST municipality deletion and one AST sampling-cluster deletion reached the random-variance boundary. Original participants and covariates were not excluded according to diagnostic results. Thyroid deletion analyses considered participants and municipalities only (Table S12).

Supplementary Figures

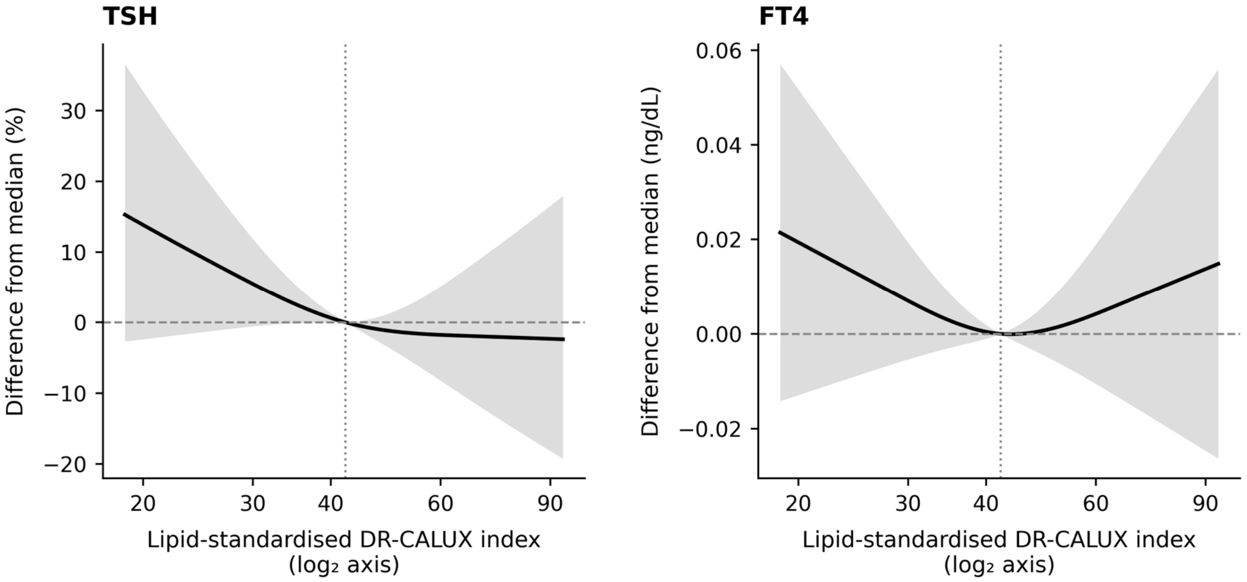

Figure S1. Exploratory TSH and FT4 exposure splines. Curves are contrasts relative to the common exposure-qualified-population median of the primary lipid-standardised index; shaded bands are pointwise 95% confidence intervals. The horizontal axis is logarithmic and covers the first–99th percentile range. The curves represent pooled adjusted contrasts. Global and nonlinear tests are reported in Table S10; neither met its applicable BH threshold.

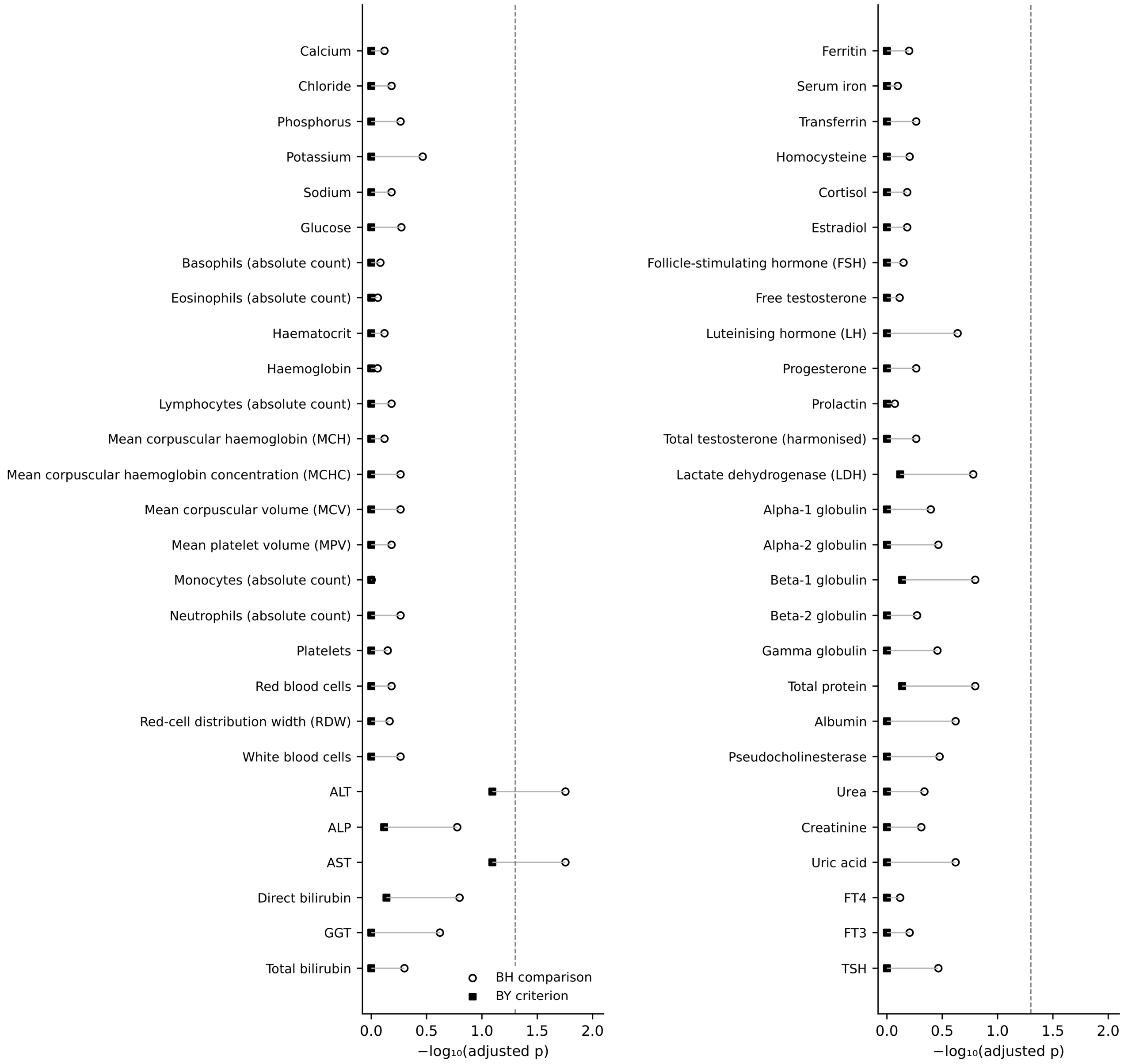

Figure S2. Complete global multiplicity screen for all 54 outcomes. Filled squares show BY-adjusted p values and open circles the BH comparison, plotted as $-\log_{10}$(adjusted p). The dashed line marks adjusted p = 0.05. No square exceeds the threshold; ALT and AST alone exceed it under BH. Positions convey adjusted statistical evidence, not effect magnitude or clinical importance. Full estimates, units and intervals are in Table S6. Endpoints are grouped by source domain and continue from the left to right panel.

References

3. Garrison, P.M.; Tullis, K.; Aarts, J.M.M.J.G.; Brouwer, A.; Giesy, J.P.; Denison, M.S. Species-specific recombinant cell lines as bioassay systems for the detection of 2,3,7,8-tetrachlorodibenzo-p-dioxin-like chemicals. Fundam. Appl. Toxicol. 1996, 30, 194–203. https://doi.org/10.1006/faat.1996.0056

4. Murk, A.J.; Leonards, P.E.G.; Bulder, A.S.; Jonas, A.S.; Rozemeijer, M.J.C.; Denison, M.S.; Koeman, J.H.; Brouwer, A. The CALUX (chemical-activated luciferase expression) assay adapted and validated for measuring TCDD equivalents in blood plasma. Environ. Toxicol. Chem. 1997, 16, 1583–1589. https://doi.org/10.1002/etc.5620160804

17. Schisterman, E.F.; Whitcomb, B.W.; Buck Louis, G.M.; Louis, T.A. Lipid adjustment in the analysis of environmental contaminants and human health risks. Environ. Health Perspect. 2005, 113, 853–857. https://doi.org/10.1289/ehp.7640

18. O'Brien, K.M.; Upson, K.; Cook, N.R.; Weinberg, C.R. Environmental Chemicals in Urine and Blood: Improving Methods for Creatinine and Lipid Adjustment. Environ. Health Perspect. 2016, 124, 220–227. https://doi.org/10.1289/ehp.1509693

19. Pierri, B.; Buonerba, C.; Coppola, A.; Pizzolante, A.; Di Stasio, A.; Cerino, P. Population-based human biomonitoring in the 'Land of Fires' area: Innovations in study design and procedures. Future Sci. OA 2021, 7, FSO646. https://doi.org/10.2144/fsoa-2020-0164

20. Pierri, B.; Buonerba, C.; Pierri, A.; Pizzolante, A.; Ferro, A.; Crispo, A.; Bollati, V.; Sanchez, T.R.; Andreassi, M.G.; Esposito, M.; Cerino, P. Exposure study on susceptible people – SPES: An integrative biomonitoring approach. Environment International 2022, 158, 106931. https://doi.org/10.1016/j.envint.2021.106931

21. Cerino, P.; Pizzolante, A.; Buonerba, C.; Montuori, P. Serum trace metals across environmental-pressure strata in Campania, Italy (SPES): a population-based cross-sectional biomonitoring study. Future Sci. OA 2026, 12, 2668083. https://doi.org/10.1080/20565623.2026.2668083

29. Li, D.; Longnecker, M.P.; Dunson, D.B. Lipid adjustment for chemical exposures: accounting for concomitant variables. Epidemiology 2013, 24, 921–928. https://doi.org/10.1097/ede.0b013e3182a671e4

30. Regione Campania; Istituto Zooprofilattico Sperimentale del Mezzogiorno. Attività di medicina traslazionale per il biomonitoraggio in Regione Campania. Technical Report; n.d. Available online: https://spes.izsmportici.it/wp-content/uploads/2021/06/Relazione-SPES.pdf (accessed on 12 September 2026).

36. Benjamini, Y.; Hochberg, Y. Controlling the false discovery rate: A practical and powerful approach to multiple testing. Journal of the Royal Statistical Society Series B 1995, 57, 289–300. https://doi.org/10.1111/j.2517-6161.1995.tb02031.x

37. Benjamini, Y.; Yekutieli, D. The control of the false discovery rate in multiple testing under dependency. Ann. Stat. 2001, 29, 1165–1188. https://doi.org/10.1214/aos/1013699998

41. Akins, J.R.; Waldrep, K.; Bernert, J.T., Jr. The estimation of total serum lipids by a completely enzymatic 'summation' method. Clin. Chim. Acta 1989, 184, 219–226. https://doi.org/10.1016/0009-8981(89)90054-5

42. Bernert, J.T.; Turner, W.E.; Patterson, D.G., Jr.; Needham, L.L. Calculation of serum "total lipid" concentrations for the adjustment of persistent organohalogen toxicant measurements in human samples. Chemosphere 2007, 68, 824–831. https://doi.org/10.1016/j.chemosphere.2007.02.043

43. VanderWeele, T.J. Principles of confounder selection. European Journal of Epidemiology 2019, 34, 211–219. https://doi.org/10.1007/s10654-019-00494-6

44. Kenward, M.G.; Roger, J.H. Small sample inference for fixed effects from restricted maximum likelihood. Biometrics 1997, 53, 983–997. https://doi.org/10.2307/2533558

45. Halekoh, U.; Højsgaard, S. A Kenward–Roger approximation and parametric bootstrap methods for tests in linear mixed models – The R package pbkrtest. Journal of Statistical Software 2014, 59, 1–32. https://doi.org/10.18637/jss.v059.i09

46. Holm, S. A simple sequentially rejective multiple test procedure. Scandinavian Journal of Statistics 1979, 6, 65–70. https://www.jstor.org/stable/4615733

47. Pustejovsky, J.E.; Tipton, E. Small-sample methods for cluster-robust variance estimation and hypothesis testing in fixed effects models. Journal of Business & Economic Statistics 2018, 36, 672–683. https://doi.org/10.1080/07350015.2016.1247004
